# Stochastic Rumor Propagation Dynamics with Log-Normal Ornstein–Uhlenbeck Process

**DOI:** 10.3390/e28101100

**Published:** 2026-10-09

**Authors:** Nafisa Ablat, Shuzhen Yu, Haijun Jiang

**Affiliations:** School of Mathematics Science, Xinjiang Normal University, Urumqi 830017, China; 15349981455@163.com (N.A.); jianghaijunxju@163.com (H.J.)

**Keywords:** rumor spreading, Ornstein–Uhlenbeck process, ergodic stationary distribution, exponential extinction

## Abstract

Considering the inevitable impact of environmental interference on rumor propagation, this paper constructs and studies a stochastic rumor propagation model in which the diffusion rate is described by the Log-normal Ornstein–Uhlenbeck (OU) process. Firstly, we rigorously prove the existence and uniqueness of global positive solutions for the model, which serves as a fundamental prerequisite for further dynamical analysis. Subsequently, by constructing an appropriate Lyapunov function and combining it with the ergodic property of the OU process, sufficient criteria for the existence of a stationary distribution of the system are given. Finally, we demonstrate that when $R_{0}^{e}\leq 1$, rumors will tend to become extinct at an exponential rate.

## 1. Introduction

Rumors refer to unverified, often misleading, and inflammatory information that can quickly spread in a short period of time. The dissemination of such information can easily trigger public panic and misunderstanding and may even disrupt social stability [1,2]. Under conditions of information asymmetry, the public often finds it difficult to accurately identify the authenticity of information and thus is more prone to trust and disseminate unverified content [3]. In this context, how to effectively curb the spread of rumors and false information while ensuring the free flow of information has become an important issue facing social governance. From a broader perspective, rumor propagation can be viewed as a self-organizing process, where macroscopic patterns—such as rumor persistence or extinction—emerge spontaneously from microscopic individual interactions, a concept discussed in the context of general dynamic systems [4].

In recent years, mathematical models have been widely applied in the study of rumor propagation mechanisms [5,6,7,8]. For example, Bernard and Pietrus [9] proposed a deterministic rumor model, in which individuals who are newly exposed to rumors are initially in an unknown state and then enter a hesitant stage after obtaining information

(1) $$\begin{array}{c} \left\{\begin{array}{cc} \frac{dI(t)}{dt} & =N-I\left(t\right)({\bar{\alpha }}_{1}S\left(t\right)+{\bar{\alpha }}_{2}U\left(t\right))-\mu I\left(t\right),\\ \frac{dR(t)}{dt} & =I\left(t\right)\left({\bar{\alpha }}_{1}S\left(t\right)+{\bar{\alpha }}_{2}U\left(t\right)\right)-\left(\beta_{1}+\beta_{2}+\mu \right)E\left(t\right),\\ \frac{dS(t)}{dt} & =\beta_{1}E\left(t\right)-\left(\theta +\mu \right)S\left(t\right),\\ \frac{dU(t)}{dt} & =\beta_{2}E\left(t\right)+\theta S\left(t\right)-\mu U\left(t\right). \end{array}\right. \end{array}$$

This model divides the total population into four categories: Ignorant (I), Hesitant (R), Spreader (S), and Opponent (U). A new individual joins the system with probability *N* as an ignorant. When ignorant individuals come into contact with Spreaders and opponents, the corresponding transmission rates are ${\bar{\alpha }}_{1}$ and ${\bar{\alpha }}_{2}$, respectively; through such contacts, ignorant individuals enter the hesitant stage. After coming into contact with a spreader or an opponent, the hesitant are further transformed into a Spreader with probability $\beta_{1}$ or into an opponent with probability $\beta_{2}$. The Spreader can also transform into an opponent with probability θ. All individuals leave the model at a rate of μ. By the next-generation matrix method, the basic reproduction number is given by

$$R_{0}=\frac{N}{\mu }\left[\frac{\alpha_{1}\beta_{1}}{\left(\beta +\mu )(\theta +\mu \right)}+\frac{\alpha_{2}\beta_{2}}{\mu (\beta +\mu )}+\frac{\alpha_{2}\theta \beta_{1}}{\mu (\beta +\mu )(\theta +\mu )}\right],\;\beta =\beta_{1}+\beta_{2}.$$

In addition, intense discussions on the spread of rumors are still ongoing in fields such as real-world society and complex networks [10,11]. However, these researches are mainly based on deterministic environments and ignore the influence of stochastic factors. In fact, during the spread of rumors, stochastic factors such as fluctuations in the status of network users and network signal interruptions inevitably affect the transmission process [12]. It is worth noting that stochastic perturbations are fundamental phenomena in the real world. The actual case presented in reference [13] shows that rumor propagation often exhibits multimodal characteristics and stochastic fluctuations. Therefore, it is more reasonable to employ stochastic differential equations for modeling rumor propagation, and several prominent stochastic rumor models have been reported in [14,15]. These research have concluded that stochastic factors cannot be ignored in rumor propagation, and it is essential to reveal how stochastic noise influences the dynamic process of rumor propagation. In many real-world scenarios, rumor propagation exhibits not only temporal fluctuations but also spatial heterogeneity, such as differences in information diffusion across regions, network topologies, or social groups [16,17,18,19]. This spatial dimension can significantly influence the speed, range, and persistence of information dissemination.

To better fit the actual propagation scenario, researchers often introduce stochastic perturbations into the model parameters through two classical approaches. The first approach models the transmission rate as a Gaussian white noise process: $\alpha \left(t\right)=\bar{\alpha }+\sigma \frac{dB(t)}{dt},$ where B(t) denotes a standard Brownian motion defined on a complete probability space $\left\{\Omega ,F,{\left\{F_{t}\right\}}_{t\geq 0},P\right\}$ with a filtration ${\left\{F_{t}\right\}}_{t\geq 0}$ and $\sigma^{2}$ is the intensity of B(t).

(2) $$\begin{array}{c} \bar{\bar{\alpha }}\left(t\right)=\frac{1}{t}\int_{0}^{t}\alpha \left(\tau \right)d\tau =\bar{\alpha }+\frac{\sigma B(t)}{t}∼N\left(\bar{\alpha },\frac{\sigma^{2}}{t}\right), \end{array}$$

this model implies that the mean of α(t) will undergo drastic changes in a remarkably short time, which is difficult to maintain for a long time as the rumor continues to spread. Although this method can effectively simulate the small random fluctuations that persist in the environment, it may lead to unreasonable short-term fluctuations during the early stage of rumor outbreak or at critical transmission nodes. This distortion fluctuation can mask the true dynamic trend of transmission, leading to an underestimation of the potential severity of the rumor transmission [20].

The second prevalent approach posits that the transmission rate follows the Ornstein–Uhlenbeck (OU) process, $d\alpha \left(t\right)=\lambda [\bar{\alpha }-\alpha \left(t\right)]dt+\sigma dB\left(t\right),$ where λ denotes the speed of reversal and σ represents the volatility of fluctuations; $\bar{\alpha }$ denotes the mean reversion level of α(t). Then

(3) $$\bar{\bar{\alpha }}\left(t\right)=\frac{1}{t}\int_{0}^{t}\alpha \left(\tau \right)d\tau =\bar{\alpha }+\frac{1}{t}\int_{0}^{t}\frac{\sigma }{\lambda }\left(1-e^{\lambda (\tau -t)}\right)dB\left(\tau \right)∼N\left(\bar{\alpha },\frac{\sigma^{2}t}{3}+o\left(t^{2}\right)\right).$$

Obviously, $E\left(\alpha \left(t\right)\right)=\bar{\alpha }$, Var(α(t))→0 as t→0; therefore, the OU process is more suitable for introduction into deterministic model (1). It can better describe the stability of parameters over a long-term range through its mean-reverting characteristic [21,22,23,24,25,26]. However, under this assumption, due to the unrestricted range of the random term’s values, the rumor transmission rate may present negative values in the mathematical derivation process, which contradicts the essential attribute of the transmission rate as a non-negative parameter. To effectively avoid this critical issue, we innovatively consider setting $\alpha_{i}\left(t\right)$(i=1,2) as a log-normal OU process. Specifically, we assume that the logarithm of the transmission rate, denoted by $X_{i}\left(t\right)=\ln\alpha_{i}\left(t\right)$, follows the OU process

$$dX_{i}\left(t\right)=\lambda_{i}\left(\ln{\bar{\alpha }}_{i}-X_{i}\left(t\right)\right)dt+\sigma_{i}dB_{i}\left(t\right),\;i=1,2.$$

Equivalently, the transmission rate itself satisfies the stochastic differential equation

$$d\alpha_{i}\left(t\right)=\alpha_{i}\left(t\right)\left[\lambda_{i}\left(\ln{\bar{\alpha }}_{i}-\ln\alpha_{i}\left(t\right)\right)+\frac{1}{2}\sigma_{i}^{2}\right]dt+\sigma_{i}\alpha_{i}\left(t\right)dB_{i}\left(t\right),$$

which can be derived by applying Itô’s formula to $X_{i}\left(t\right)=\ln\alpha_{i}\left(t\right)$. Indeed, let $f\left(x\right)=e^{x}$, so that $\alpha_{i}=f\left(X_{i}\right)$. By Itô’s formula,

$$d\alpha_{i}=f^{\prime }\left(X_{i}\right)dX_{i}+\frac{1}{2}f^{\prime \prime }\left(X_{i}\right){\left(dX_{i}\right)}^{2}=\alpha_{i}dX_{i}+\frac{1}{2}\alpha_{i}\sigma_{i}^{2}dt,$$

which, after substituting $dX_{i}$, yields the equation above. Since $\alpha_{i}\left(t\right)=e^{X_{i}\left(t\right)}$ is an exponential function of a Gaussian process, $\alpha_{i}\left(t\right)$ is log-normally distributed at each time *t*, and remains strictly positive for all t≥0. The explicit solution of the log-normal OU process is given by

$$\alpha_{i}\left(t\right)={\bar{\alpha }}_{i}\exp\left(e^{-\lambda_{i}t}\ln\frac{\alpha_{i}\left(0\right)}{{\bar{\alpha }}_{i}}+\sigma_{i}\int_{0}^{t}e^{-\lambda_{i}\left(t-s\right)}dB_{i}\left(s\right)\right),$$

from which one can further derive its expectation and variance. Unlike the conventional white noise perturbation, which assumes that the transmission rate fluctuates around a constant with unbounded variance in the short term, the log-normal OU process introduces a mean-reverting mechanism. This implies that the transmission rates $\alpha_{1}\left(t\right)$ and $\alpha_{2}\left(t\right)$ tend to revert to their long-term average levels ${\bar{\alpha }}_{1}$ and ${\bar{\alpha }}_{2}$ over time, with the speed of reversion governed by $\lambda_{1}$ and $\lambda_{2}$. Such a property is particularly suitable for rumor propagation, because in reality, the public’s susceptibility and willingness to spread rumors do not change arbitrarily in the very short term; instead, they exhibit stable tendencies with occasional random shocks. Moreover, by taking the logarithmic transformation, we ensure that $\alpha_{i}\left(t\right)$ remains strictly positive, which is consistent with the physical meaning of transmission rates. This combination of positivity and mean reversion makes the log-normal OU process a more realistic choice for modeling stochastic fluctuations in rumor dynamics compared to the classical white noise approach. While retaining the mean-reverting characteristics of the OU process, proposed the following more realistic stochastic rumor propagation model:

(4) $$\begin{array}{c} \left\{\begin{array}{c} \frac{dI(t)}{dt}=N-I\left(t\right)\left({\bar{\alpha }}_{1}S\left(t\right)+{\bar{\alpha }}_{2}U\left(t\right)\right)-\mu I\left(t\right),\\ \frac{dE(t)}{dt}=I\left(t\right)\left({\bar{\alpha }}_{1}S\left(t\right)+{\bar{\alpha }}_{2}U\left(t\right)\right)-\left(\beta +\mu \right)E\left(t\right),\\ \frac{dS(t)}{dt}=\beta_{1}E\left(t\right)-\left(\theta +\mu \right)S\left(t\right),\\ \frac{dU(t)}{dt}=\beta_{2}E\left(t\right)+\theta S\left(t\right)-\mu U\left(t\right),\\ d\ln\alpha_{1}=\lambda_{1}\left(\ln{\bar{\alpha }}_{1}-\ln\alpha_{1}\right)dt+\sigma_{1}\;dB_{1}\left(t\right),\\ d\ln\alpha_{2}=\lambda_{2}\left(\ln{\bar{\alpha }}_{2}-\ln\alpha_{2}\right)dt+\sigma_{2}\;dB_{2}\left(t\right), \end{array}\right. \end{array}$$

where $\beta =\beta_{1}+\beta_{2}$. The first four equations of (4) follow the framework of [9] in the deterministic case. The main extension here is that the transmission rates $\alpha_{1}\left(t\right)$ and $\alpha_{2}\left(t\right)$ are modeled as log-normal Ornstein–Uhlenbeck processes.

The main contributions of this paper are threefold:

- Existing stochastic rumor models predominantly employ Gaussian white noise perturbations, which exhibit infinite instantaneous variance and may lead to negative transmission rates—both mathematically and physically objectionable. In this paper, we introduce, for the first time, the log-normal Ornstein–Uhlenbeck process into rumor propagation models. This approach ensures that transmission rates remain strictly positive. Meanwhile, it captures, via the mean-reverting property, the inherent tendency of the information environment to return to equilibrium after random shocks, thereby overcoming the fundamental drawbacks of conventional modeling frameworks.
- For the proposed model, we not only prove the existence and uniqueness of a global positive solution but, more importantly, derive two computable critical thresholds—the stationary-distribution threshold $R_{0}^{s}$ and the extinction threshold $R_{0}^{e}$, which are stochastic analogues of the basic reproduction number $R_{0}$ of the deterministic model (1), and Section 3 and Section 4 for the definitions of $R_{0}^{s}$ and $R_{0}^{e}$, respectively. We rigorously establish that when $R_{0}^{s}>1$, the system admits a stationary distribution, and when $R_{0}^{e}<1$, rumor spreaders tend to extinction almost surely with an exponential decay rate. This dual-threshold criterion offers a full theoretical characterization of the long-term dynamics of rumor propagation.
- Both theoretical analysis and numerical simulations consistently reveal that $R_{0}^{s}$ and $R_{0}^{e}$ are jointly modulated by stochastic fluctuations. Since $R_{0}^{s}\geq R_{0}$, noise tends to sustain rumor persistence; since $R_{0}^{e}\geq R_{0}$, noise also makes extinction harder. However, reducing the noise intensity $\sigma_{1},\sigma_{2}$ or increasing the mean-reversion speed $\lambda_{1},\lambda_{2}$ lowers both thresholds simultaneously and drives rumor spreaders toward extinction.

The structure of this paper is as follows: in Section 2, the existence and uniqueness of global positive solutions are proven. The existence of a stationary distribution for model (4) is proved by constructing an appropriate Lyapunov function in Section 3, Section 4 provides sufficient conditions for the extinction of rumor. Section 5 verifies the analysis results of the previous sections through numerical simulations and ultimately draws conclusions.

## 2. Existence and Uniqueness of a Global Solution

In this section, we first give the existence and global positive property of the partial solution (I(t),E(t),S(t),U(t)) to model (4), laying the foundation for subsequent analysis.

Before proceeding to the existence and uniqueness of the global positive solution, we first introduce the infinitesimal generator associated with the stochastic model (4). Let $X\left(t\right)={\left(I\left(t\right),E\left(t\right),S\left(t\right),U\left(t\right),\alpha_{1}\left(t\right),\alpha_{2}\left(t\right)\right)}^{T}$ and rewrite model (4) in the general form

dX(t)=f(X(t))dt+g(X(t))dB(t),

where $B\left(t\right)={\left(B_{1}\left(t\right),B_{2}\left(t\right)\right)}^{T}$ is a two-dimensional standard Brownian motion, $f:R_{+}^{6}\to R^{6}$ is the drift vector, and $g:R_{+}^{6}\to R^{6\times 2}$ is the diffusion matrix. For a $C^{2}$ function $V:R_{+}^{6}\to R$, the infinitesimal generator L acting on *V* is defined by

$$LV\left(x\right)=\sum_{i=1}^{6}f_{i}\left(x\right)\frac{\partial V(x)}{\partial x_{i}}+\frac{1}{2}\sum_{i,j=1}^{6}{\left(gg^{T}\right)}_{ij}\left(x\right)\frac{\partial^{2}V\left(x\right)}{\partial x_{i}\partial x_{j}}.$$

Equivalently, by Itô’s formula, LV(x) is the drift term of V(X(t)), i.e.,

$$dV\left(X\left(t\right)\right)=LV\left(X\left(t\right)\right)dt+\sum_{i=1}^{6}\sum_{j=1}^{2}g_{ij}\left(X\left(t\right)\right)\frac{\partial V(X(t))}{\partial x_{i}}dB_{j}\left(t\right).$$

**Theorem** **1.** *For any initial value $\left(I\left(0\right),E\left(0\right),S\left(0\right),U\left(0\right),\alpha_{1}\left(0\right),\alpha_{2}\left(0\right)\right)\in R_{+}^{6}$, model (4) possesses a unique global positive solution $\left(I\left(t\right),E\left(t\right),S\left(t\right),U\left(t\right),\alpha_{1}\left(t\right),\alpha_{2}\left(t\right)\right)$ for all t≥0 with probability one (a.s.).*

**Proof.**  It is obvious that all coefficients of system (4) satisfy the Lipschitz continuity, which implies that system (4) admits a unique solution $\left(I(t),E(t),S(t),U(t),\alpha (t)\right)$ on $t\in [0,\tau_{e}]$, where $\tau_{e}$ denotes the explosion time. To prove the global existence of the solution, it suffices to show that $\tau_{e}=+\infty$ almost surely. Let $\rho_{0}$ be a positive integer such that each component of $\left(I\left(0\right),E\left(0\right),S\left(0\right),U\left(0\right),\alpha_{1}\left(0\right),\alpha_{2}\left(0\right)\right)\in R_{+}^{6}$ lies in $\left[\frac{1}{\rho_{0}},\rho_{0}\right]$. For every $\rho >\rho_{0}$, define a stopping time

$$\tau_{\rho }=\inf\left\{0<t<\tau_{e}\;|\;\begin{array}{c} \min\left\{I\left(t\right),E\left(t\right),S\left(t\right),U\left(t\right),\alpha_{1}\left(t\right),\alpha_{2}\left(t\right)\right\}<\frac{1}{\rho }\;\mathrm{or}\\ \max\left\{I\left(t\right),E\left(t\right),S\left(t\right),U\left(t\right),\alpha_{1}\left(t\right),\alpha_{2}\left(t\right)\right\}>\rho \end{array}\right\}.$$

We set infØ=+∞, where Ø stands for an empty set. As ρ→+∞, $\tau_{\rho }$ is increasing. Denote $\tau_{\infty }=\lim_{\rho \to +\infty }\tau_{\rho },$ then $\tau_{\infty }\leq \tau_{\rho }$ a.s. Thus, we need to prove that $\tau_{\infty }=+\infty$ a.s. Use the method of contradiction. If not, then there exist three constants $\left(\kappa ,\rho_{1},T_{0}\right)\in \left(\left(0,1\right),N^{*},R^{+}\right)$ such that for any $\rho_{1}\geq \rho$,

(5) $$P(\tau_{\rho }\leq T_{0})\geq \kappa .$$

To prove the non-explosion property, we construct a nonnegative $C^{2}$ Lyapunov function [27]. based on the elementary inequality x−1−lnx≥0 for x>0. The logarithmic terms $-\ln I,-\ln E,\ldots ,-\ln\alpha_{2}$ ensure that Q→+∞ as any component approaches zero or infinity, which is the key to ruling out finite-time explosion. Specifically, define $Q\left(I,E,S,U,\alpha_{1},\alpha_{2}\right):R_{+}^{6}\to R_{+}$ as follows:

$$\begin{array}{cc} Q(I,E,S,U,\alpha_{1},\alpha_{2})= & I-1-\ln I+E-1-\ln E+S-1-\ln S\\ \; & +U-1-\ln U+\alpha_{1}-1-\ln\alpha_{1}+\alpha_{2}-1-\ln\alpha_{2}.\; \end{array}$$

Applying Itô’s formula yields

(6) $$\begin{array}{cc} LQ & =N-I\left(\alpha_{1}S+\alpha_{2}U\right)-\mu I-\frac{N}{I}+\left(\alpha_{1}S+\alpha_{2}U\right)+\mu \\ \; & \;+I\left(\alpha_{1}S+\alpha_{2}U\right)-\left(\beta +\mu \right)E-\frac{I}{E}\left(\alpha_{1}S+\alpha_{2}U\right)+\beta +\mu \\ \; & \;+\beta_{2}E-\left(\theta +\mu \right)S-\frac{\beta_{1}E}{S}+\theta +\mu +\beta_{2}E-\theta S-\mu U\\ \; & \;-\frac{\beta_{2}E}{U}-\frac{\theta S}{U}+\mu +\sum_{i=1}^{2}\left[{\bar{\alpha }}_{i}\left(\lambda_{i}\left(\ln{\bar{\alpha }}_{i}-\ln\alpha_{i}\right)+\frac{1}{2}\sigma_{i}^{2}\right)-\lambda_{i}\left(\ln{\bar{\alpha }}_{i}-\ln\alpha_{i}\right)\right]\\ \; & \leq N+4\mu +\theta +\beta +\left(\alpha_{1}+\alpha_{2}\right)\frac{N}{\mu }\\ \; & \;+\sum_{i=1}^{2}\left[\bar{\alpha_{i}}\left(\lambda_{i}\left(\ln{\bar{\alpha }}_{i}-\ln\alpha_{i}\right)+\frac{1}{2}\sigma_{i}^{2}\right)-\lambda_{i}\left(\ln{\bar{\alpha }}_{i}-\ln\alpha_{i}\right)\right]. \end{array}$$

Observe that $K(\alpha_{1},\alpha_{2})\to -\infty$ as $\alpha_{1},\alpha_{2}\to +\infty$ or $\alpha_{1},\alpha_{2}\to 0$. Hence there exists a positive constant $k_{0}$ such that

(7) $$\sup\left\{\left(\alpha_{1}+\alpha_{2}\right)\frac{N}{\mu }+\sum_{i=1}^{2}\left[\alpha_{i}\left(\lambda_{i}\left(\ln{\bar{\alpha }}_{i}-\ln\alpha_{i}\right)+\frac{1}{2}\sigma_{i}^{2}\right)-\lambda_{i}\left(\ln{\bar{\alpha }}_{i}-\ln\alpha_{i}\right)\right]\right\}\leq k_{0}.$$

Then inequality (6) can be rewritten as

(8) $$LQ\leq N+4\mu +\theta +\beta +k_{0}:=K_{1}.$$

By applying the generalized Itô integral to *Q*, it follows from (8) that

$$\begin{array}{cc} 0 & \leq E\left[Q\left(I\left(\tau_{\rho }\wedge T_{0}\right),E\left(\tau_{\rho }\wedge T_{0}\right),S\left(\tau_{\rho }\wedge T_{0}\right),U\left(\tau_{\rho }\wedge T_{0}\right),\alpha_{i}\left(\tau_{\rho }\wedge T_{0}\right)\right)\right]\\ & =E\left[Q\left(I\left(0\right),E\left(0\right),S\left(0\right),U\left(0\right),\alpha_{i}\left(0\right)\right)\right]+E\int_{0}^{\tau_{\rho }\wedge T_{0}}LQ\;dt\\ & \leq E\left[Q\left(I\left(0\right),E\left(0\right),S\left(0\right),U\left(0\right),\alpha_{i}\left(0\right)\right)\right]+K_{1}T_{0}. \end{array}$$

For $\rho \geq \rho_{1}$, denote $\Omega_{\rho }=\left\{\tau_{\rho }\leq T_{0}\right\}$. From Equation (5), we have $P(\Omega_{\rho })\geq \kappa$. By the definition of stopping time: for any sample $\tau \in \Omega_{\rho }$, at least one of $I\left(\tau_{\rho }\right),E\left(\tau_{\rho }\right),S\left(\tau_{\rho }\right),U\left(\tau_{\rho }\right),\alpha_{1}\left(\tau_{\rho }\right),\alpha_{2}\left(\tau_{\rho }\right)$ equals either $\frac{1}{\rho }$ or ρ. Therefore

$$\begin{array}{cc} EQ\left(X(\tau_{\rho }\wedge T_{0})\right) & =E\left[1_{\Omega_{\rho }}Q\left(I\left(\tau_{\rho }\right),\;E\left(\tau_{\rho }\right),\;S\left(\tau_{\rho }\right),\;U\left(\tau_{\rho }\right),\;\alpha_{1}\left(\tau_{\rho }\right),\;\alpha_{2}\left(\tau_{\rho }\right)\right)\right]\\ & \geq \kappa \left[\left(\rho -1-\ln\rho \right)\;\wedge \;\left(\frac{1}{\rho }-1+\ln\rho \right)\right]. \end{array}$$

Letting ρ→+∞, we arrive at a contradiction: $+\infty >Q\left(I\left(0\right),E\left(0\right),S\left(0\right),U\left(0\right),\alpha_{1}\left(0\right),\alpha_{2}\left(0\right)\right)+K_{1}T_{0}=+\infty .$ Consequently, $\tau_{\infty }=+\infty$ holds almost surely. This completes the proof. □

**Remark** **1.** *Throughout the following sections, we maintain the assumption that the initial condition satisfies $S\left(0\right)+E\left(0\right)+I\left(0\right)+U\left(0\right)<\frac{N}{\mu }$, which means $\Omega :=\{\left(S\left(t\right),E\left(t\right),I\left(t\right),U\left(t\right),\alpha_{1}\left(t\right),\alpha_{2}\left(t\right)\right)\in R_{+}^{6}|I+E+S+U\leq \frac{N}{\mu }\}$ is identified as the invariant set of the model (4).*

## 3. Stationary Distribution

In the analysis of stochastic rumor models, the stationary distribution is essential for studying long-term persistence. This section is devoted to deriving sufficient conditions that guarantee the existence of stationary distribution. Define

$$R_{0}^{s}=\frac{N}{\mu }\left[\frac{{\tilde{\alpha }}_{1}\beta_{1}}{\left(\beta +\mu )(\theta +\mu \right)}+\frac{{\tilde{\alpha }}_{2}\beta_{2}}{\mu (\beta +\mu )}+\frac{{\tilde{\alpha }}_{2}^{\prime }\theta \beta_{1}}{\mu (\beta +\mu )(\theta +\mu )}\right],$$

where ${\tilde{\alpha }}_{1}={\bar{\alpha }}_{1}e^{\frac{\sigma_{1}^{2}}{12\lambda_{1}}},\;{\tilde{\alpha }}_{2}={\bar{\alpha }}_{2}e^{\frac{\sigma_{2}^{2}}{12\lambda_{2}}},\;{{\tilde{\alpha }}_{2}}^{\prime }={\bar{\alpha }}_{2}e^{\frac{\sigma_{2}^{2}}{16\lambda_{2}}}.$

**Theorem** **2.** *If $R_{0}^{s}>1$, model (4) admits at least one stationary distribution.*

**Proof.**  We propose to verify the theorem through a two-step approach.

Step I (Establishment of suitable Lyapunov functions)

By It$\hat{o}$ formula, we define $V_{1}=-\ln E-\left(c_{1}+c_{2}\right)\ln I-c_{3}\ln S-c_{4}\ln U,$ where $c_{i}$ (i=1,2,3,4) are presented in the subsequent proof. Applying Itô’s formula to $V_{1}$ and using the arithmetic mean-geometric mean (AM–GM) inequality $a+b+c\geq 3\sqrt[{3}]{abc}$, we get

$$\begin{array}{cc} LV_{1}= & -\frac{\alpha_{1}SI}{E}-\frac{\alpha_{2}UI}{E}+\left(\beta +\mu \right)-\frac{c_{1}N}{I}+c_{1}\left(\alpha_{1}S+\alpha_{2}U\right)+c_{1}\mu -\frac{c_{2}N}{I}+c_{2}\left(\alpha_{1}S+\alpha_{2}U\right)\\ \; & +c_{2}\mu -\frac{c_{3}\beta_{1}E}{S}+c_{3}\left(\theta +\mu \right)-\frac{c_{4}\beta_{2}E}{U}-\frac{c_{4}\theta S}{U}+c_{4}\mu \\ \leq & -3\sqrt[{3}]{N{\tilde{\alpha }}_{1}\beta_{1}c_{1}c_{3}}-3\sqrt[{3}]{N{\tilde{\alpha }}_{2}\beta_{2}c_{2}c_{4}}+\beta +\mu +\left(c_{1}+c_{2}\right)\left(\alpha_{1}S+\alpha_{2}U\right)\\ \; & +3\left(\sqrt[{3}]{N{\tilde{\alpha }}_{1}\beta_{1}c_{1}c_{3}}-\sqrt[{3}]{N\alpha_{1}\beta_{1}c_{1}c_{3}}\right)+3\left(\sqrt[{3}]{N{\tilde{\alpha }}_{2}\beta_{2}c_{2}c_{4}}-\sqrt[{3}]{N\alpha_{2}\beta_{2}c_{2}c_{4}}\right). \end{array}$$

To enable the combination of these negative terms, let $c_{1},c_{2},c_{3}$ and $c_{4}$ satisfy the following equations

(9) $$\begin{array}{cc} c_{1}\mu & =c_{3}\left(\theta +\mu \right)=\frac{N{\tilde{\alpha }}_{1}\beta_{1}}{\mu (\theta +\mu )},\;\;c_{2}\mu =c_{4}\mu =\frac{N{\tilde{\alpha }}_{2}\beta_{2}}{\mu^{2}}. \end{array}$$

Then $LV_{1}$ turns into

(10) $$\begin{array}{cc} LV_{1} & \leq -\frac{N{\tilde{\alpha }}_{1}\beta_{1}}{\mu (\theta +\mu )}-\frac{N{\tilde{\alpha }}_{2}\beta_{2}}{\mu^{2}}+\beta +\mu +\left(c_{1}+c_{2}\right)\left(\alpha_{1}S+\alpha_{2}U\right)\\ \; & \;+3\left(\sqrt[{3}]{N{\tilde{\alpha }}_{1}\beta_{1}c_{1}c_{3}}-\sqrt[{3}]{N\alpha_{1}\beta_{1}c_{1}c_{3}}+\right)+3\left(\sqrt[{3}]{N{\tilde{\alpha }}_{2}\beta_{2}c_{2}c_{4}}-\sqrt[{3}]{N\alpha_{2}\beta_{2}c_{2}c_{4}}\right). \end{array}$$

Next, define $V_{2}=V_{1}-\ln E-c_{5}\ln S-c_{6}\ln U-c_{7}\ln I$, where $c_{i}$ (i=5,6,7) are presented in the following proof. Applying Itô’s formula to the function $V_{2}$, we get

(11) $$\begin{array}{cc} LV_{2}\leq & -\frac{N{\tilde{\alpha }}_{1}\beta_{1}}{\mu (\theta +\mu )}-\frac{N{\tilde{\alpha }}_{2}\beta_{2}}{\mu^{2}}+\beta +\mu +\left(c_{1}+c_{2}\right)\left(\alpha_{1}S+\alpha_{2}U\right)-\frac{\alpha_{1}SI}{E}-\frac{\alpha_{2}UI}{E}+\mu +\beta \\ \; & -\frac{c_{5}\beta_{1}E}{S}+c_{5}\left(\theta +\mu \right)-\frac{c_{6}\beta_{2}E}{U}-\frac{c_{6}\theta S}{U}+c_{6}\mu -\frac{c_{7}N}{I}+c_{7}\left(\alpha_{1}S+\alpha_{2}U\right)+c_{7}\mu \\ \; & +3\left(\sqrt[{3}]{N{\tilde{\alpha }}_{1}\beta_{1}c_{1}c_{3}}-\sqrt[{3}]{N\alpha_{1}\beta_{1}c_{1}c_{3}}\right)+3\left(\sqrt[{3}]{N{\tilde{\alpha }}_{2}\beta_{2}c_{2}c_{4}}-\sqrt[{3}]{N\alpha_{2}\beta_{2}c_{2}c_{4}}\right). \end{array}$$

Let $c_{5},c_{6},c_{7}$ satisfy the following equations

(12) $$\begin{array}{c} c_{5}\left(\theta +\mu \right)=c_{6}\mu =c_{7}\mu =\frac{\tilde{\alpha_{2}}\beta_{2}\theta N}{\mu^{2}\left(\theta +\mu \right)}, \end{array}$$

then $LV_{2}$ turns into

$$\begin{array}{cc} LV_{2}\leq & -\left(\beta +\mu \right)\left(R_{0}^{s}-1\right)+\left(c_{1}+c_{2}+c_{7}\right)\left(\alpha_{1}S+\alpha_{2}U\right)+\mu +\beta \\ \; & +3\left(\sqrt[{3}]{N{\tilde{\alpha }}_{1}\beta_{1}c_{1}c_{3}}-\sqrt[{3}]{N\alpha_{1}\beta_{1}c_{1}c_{3}}+\right)+3\left(\sqrt[{3}]{N{\tilde{\alpha }}_{2}\beta_{2}c_{2}c_{4}}-\sqrt[{3}]{N\alpha_{2}\beta_{2}c_{2}c_{4}}\right)\\ \; & +4\left(\sqrt[{4}]{N{\tilde{\alpha }}_{2}^{\prime }\theta \beta_{1}c_{5}c_{6}c_{7}}-\sqrt[{4}]{N\alpha_{2}\theta \beta_{1}c_{5}c_{6}c_{7}}\right). \end{array}$$

Since $R_{0}^{s}>1$, there exists a sufficiently small positive constant ω such that

(13) $$\begin{array}{c} R_{0}^{s}\left(\omega \right)≜R_{0}^{s}-\frac{\left(c_{1}+c_{2}+c_{7}\right)I_{0}\omega }{\mu +\beta }\left({\hat{\alpha }}_{1}+{\hat{\alpha }}_{2}\right)>1, \end{array}$$

where ${\hat{\alpha }}_{1}={\bar{\alpha }}_{1}^{2}e^{\frac{\sigma_{1}^{2}}{\lambda_{1}}}$ and ${\hat{\alpha }}_{2}={\bar{\alpha }}_{2}^{2}e^{\frac{\sigma_{2}^{2}}{\lambda_{2}}}$. By the inequality $x\leq ax^{2}+\frac{1}{4a}$ (a>0), we have

(14) $$\begin{array}{c} \alpha_{1}S\leq \left(\omega \alpha_{1}^{2}+\frac{1}{4\omega }\right)S\leq \omega I_{0}\alpha_{1}^{2}+\frac{1}{4\omega }S=\omega I_{0}{\hat{\alpha }}_{1}+\frac{1}{4\omega }S+\omega I_{0}\left(\alpha_{1}^{2}-{\hat{\alpha }}_{1}\right),\\ \alpha_{2}U\leq \left(\omega \alpha_{2}^{2}+\frac{1}{4\omega }\right)U\leq \omega I_{0}\alpha_{2}^{2}+\frac{1}{4\omega }U=\omega I_{0}{\hat{\alpha }}_{2}+\frac{1}{4\omega }U+\omega I_{0}\left(\alpha_{2}^{2}-{\hat{\alpha }}_{2}\right). \end{array}$$

Combining inequalities (14), the inequality (10) becomes

$$\begin{array}{cc} LV_{2}\leq & -\left(\beta +\mu \right)\left(R_{0}^{s}-1\right)+\left(c_{1}+c_{2}+c_{7}\right)\left[\omega I_{0}{\hat{\alpha }}_{1}+\frac{1}{4\omega }S+\omega I_{0}\left(\alpha_{1}^{2}-{\hat{\alpha }}_{1}\right)\right]+\mu +\beta \\ \; & +\left(c_{1}+c_{2}+c_{7}\right)\left[\omega I_{0}{\hat{\alpha }}_{2}+\frac{1}{4\omega }U+\omega I_{0}\left(\alpha_{2}^{2}-{\hat{\alpha }}_{2}\right)\right]\\ \; & +3\left(\sqrt[{3}]{N\tilde{\alpha_{1}}\beta_{1}c_{1}c_{3}}-\sqrt[{3}]{N\alpha_{1}\beta_{1}c_{1}c_{3}}+\right)+3\left(\sqrt[{3}]{N\tilde{\alpha_{2}}\beta_{2}c_{2}c_{4}}-\sqrt[{3}]{N\alpha_{2}\beta_{2}c_{2}c_{4}}\right)\\ \; & +4\left(\sqrt[{4}]{N{\tilde{\alpha }}_{2}^{\prime }\theta \beta_{1}c_{5}c_{6}c_{7}}-\sqrt[{4}]{N\alpha_{2}\theta \beta_{1}c_{5}c_{6}c_{7}}\right)\\ = & -\left(\beta +\mu \right)[R_{0}^{s}\left(\omega \right)-1]+\frac{\left(c_{1}+c_{2}+c_{7}\right)}{4\omega }S+\frac{\left(c_{1}+c_{2}+c_{7}\right)}{4\omega }U+H\left(\alpha_{1},\alpha_{2}\right), \end{array}$$

where

$$\begin{array}{cc} H(\alpha_{1},\alpha_{2})= & \mu +\beta +\left(c_{1}+c_{2}+c_{7}\right)\omega I_{0}\left[\left(\alpha_{1}^{2}-{\hat{\alpha }}_{1}\right)+\left(\alpha_{2}^{2}-{\hat{\alpha }}_{2}\right)\right]\\ \; & +3\left(\sqrt[{3}]{N\tilde{\alpha_{1}}\beta_{1}c_{1}c_{3}}-\sqrt[{3}]{N\alpha_{1}\beta_{1}c_{1}c_{3}}\right)+3\left(\sqrt[{3}]{N\tilde{\alpha_{2}}\beta_{2}c_{2}c_{4}}-\sqrt[{3}]{N\alpha_{2}\beta_{2}c_{2}c_{4}}\right)\\ \; & +4(\sqrt[{4}]{N{\tilde{\alpha }}_{2}^{\prime }\theta \beta_{1}c_{5}c_{6}c_{7}}-\sqrt[{4}]{N\alpha_{2}\theta \beta_{1}c_{5}c_{6}c_{7}}). \end{array}$$

Next, we define the function $V_{3}=V_{2}+c_{8}S+c_{8}U$, where $c_{8}$ is determined later. Similarly,

$$\begin{array}{cc} LV_{3}\leq & -\left(\beta +\mu \right)[R_{0}^{s}\left(\omega \right)-1]+\left[\frac{\left(c_{1}+c_{2}+c_{7}\right)}{4\omega }-c_{8}\mu \right]S\\ \; & +\left[\frac{\left(c_{1}+c_{2}+c_{7}\right)}{4\omega }-c_{8}\mu \right]U+c_{8}\left(\beta_{1}+\beta_{2}\right)E+H\left(\alpha_{1},\alpha_{2}\right). \end{array}$$

Take $c_{8}=\frac{\left(c_{1}+c_{2}+c_{7}\right)}{4\omega \mu }$ to make the coefficient of S,U equal to 0, define $\eta =\frac{(c_{1}+c_{2}+c_{7})\beta }{4\omega \mu }$, thus

(15) $$LV_{3}\leq -\left(\beta +\mu \right)[R_{0}^{s}\left(\omega \right)-1]+\eta E+H\left(\alpha_{1},\alpha_{2}\right).$$

Let $\bar{V}=MV_{3}-\ln I-\ln E-\ln S-\ln U-\ln\left[(\frac{N}{\mu }-\left(I+E+S+U\right)\right]+\alpha_{1}-1-\ln\alpha_{1}+\alpha_{2}-1-\ln\alpha_{2},$ where *M* is a sufficiently large positive constant satisfying

(16) $$-M\left(\beta +\mu \right)\left[R_{0}^{s}\left(\omega \right)-1\right]+\underset{\left(\alpha_{1},\alpha_{2}\right)\in R_{+}^{2}}{\sup}W\left(\alpha_{1},\alpha_{2}\right)\leq -2,$$

in which

$$\begin{array}{cc} W(\alpha_{1},\alpha_{2})= & 2\mu +\theta +\frac{\alpha_{1}N}{\mu }+\frac{\alpha_{2}N}{\mu }\\ \; & +\sum_{i=1}^{2}\left[\alpha_{i}\left(\lambda_{i}\ln{\bar{\alpha }}_{i}-\frac{1}{2}\lambda_{i}\ln\alpha_{i}+\frac{1}{2}\sigma_{i}^{2}\right)-\lambda_{i}\left(\ln{\bar{\alpha }}_{i}-\frac{1}{2}\ln\alpha_{i}\right)\right]. \end{array}$$

We notice that $\bar{V}\left(I,E,S,U,\alpha_{1},\alpha_{2}\right)\to \infty$ as $\left(I,E,S,U,\alpha_{1},\alpha_{2}\right)$ approaches the boundary of Ω. Thus the continuous function $\bar{V}$ admits a minimum value ${\bar{V}}_{\min}$ within the interior of Ω. Define a state vector $\rho =(I,E,S,U,\alpha_{1},\alpha_{2})$ and a non-negative $C^{2}$-function $V:\Omega \to R_{+}$ by $V\left(\rho \right)=\bar{V}\left(\rho \right)-{\bar{V}}_{\min}.$ Applying Itô’s formula to the Lyapunov function *V* gets

(17) $$\begin{array}{cc} LV\leq & -M\left(\beta +\mu \right)[R_{0}^{s}\left(\omega \right)-1]+M\eta E-\frac{N}{I}+\frac{\alpha_{1}N}{\mu }+\frac{\alpha_{2}N}{\mu }+2\mu +\theta -\frac{\beta_{1}E}{S}\\ \; & -\frac{\theta S}{U}-\frac{\beta_{2}E}{U}+\sum_{i=1}^{2}\left[\alpha_{i}\left(\lambda_{i}\ln{\bar{\alpha }}_{i}-\lambda_{i}\ln\alpha_{i}+\frac{1}{2}\sigma_{i}^{2}\right)-\lambda_{i}\left(\ln{\bar{\alpha }}_{i}-\ln\alpha_{i}\right)\right]+MH\left(\alpha_{1},\alpha_{2}\right)\\ \leq & -M\left(\beta +\mu \right)[R_{0}^{s}\left(\omega \right)-1]+M\eta E-\frac{N}{I}-\frac{\beta_{1}E}{S}-\frac{\beta_{2}E}{U}\\ \; & -\frac{1}{2}\alpha_{1}\lambda_{1}\ln\alpha_{1}+\frac{1}{2}\lambda_{1}\ln\alpha_{1}-\frac{1}{2}\alpha_{2}\lambda_{2}\ln\alpha_{2}+\frac{1}{2}\lambda_{2}\ln\alpha_{2}+W\left(\alpha_{1},\alpha_{2}\right)+MH\left(\alpha_{1},\alpha_{2}\right)\\ \leq & G\left(I,E,S,U,\alpha_{1},\alpha_{2}\right)+MH\left(\alpha_{1},\alpha_{2}\right), \end{array}$$

where $G\left(\rho \right)=-M\left(\beta +\mu \right)[R_{0}^{s}\left(\omega \right)-1]+M\eta E-\frac{N}{I}-\frac{\beta_{1}E}{S}-\frac{\beta_{2}E}{U}-\frac{1}{2}\alpha_{1}\lambda_{1}\ln\alpha_{1}+\frac{1}{2}\lambda_{1}\ln\alpha_{1}-\frac{1}{2}\alpha_{2}\lambda_{2}\ln\alpha_{2}+\frac{1}{2}\lambda_{2}\ln\alpha_{2}+W\left(\alpha_{1},\alpha_{2}\right).$ It is obvious that $W(\alpha_{1},\alpha_{2})\to -\infty$ as $\alpha_{1}\to +\infty$, $\alpha_{1}\to 0^{+}$, $\alpha_{2}\to +\infty$ or $\alpha_{2}\to 0^{+}$, which implies $W(\alpha_{1},\alpha_{2})$ attains its supremum.

Step II (The search of a compact set $D_{\varepsilon }$)

Let us define a closed and bounded set contained in the invariant set Ω as

$$D_{\varepsilon }=\left\{\rho \in \Omega \;|\;I\geq \varepsilon ,\;E\geq \varepsilon ,\;U\geq \varepsilon^{2},\;S\geq \varepsilon^{2},\varepsilon \leq \alpha_{1}\leq \frac{1}{\varepsilon },\;\varepsilon \leq \alpha_{2}\leq \frac{1}{\varepsilon }\right\},$$

then $\Omega \setminus D_{\epsilon }={⋃}_{i=1}^{8}D_{\varepsilon }^{i}$, where

$$\begin{array}{cc} D_{\varepsilon }^{1} & =\left\{\rho \in \Omega |0<\alpha_{1}<\varepsilon \right\},D_{\varepsilon }^{2}=\left\{\rho \in \Omega |\alpha_{1}>\frac{1}{\varepsilon }\right\},D_{\varepsilon }^{3}=\left\{\rho \in \Omega |0<\alpha_{2}<\varepsilon \right\},\\ D_{\varepsilon }^{4} & =\left\{\rho \in \Omega |\alpha_{2}>\frac{1}{\varepsilon }\right\},D_{\varepsilon }^{5}=\left\{\rho \in \Omega |0<I<\varepsilon \right\},D_{\varepsilon }^{6}=\left\{\rho \in \Omega |0<E<\varepsilon \right\},\\ D_{\varepsilon }^{7} & =\left\{\rho \in \Omega |E\geq \varepsilon ,0<U<\varepsilon^{2}\right\},D_{\varepsilon }^{8}=\left\{\rho \in \Omega |E\geq \varepsilon ,0<S<\varepsilon^{2}\right\}, \end{array}$$

where ε>0 is a sufficiently small constant satisfying

(18) $$\begin{array}{cc} \; & -M\left(\beta +\mu \right)[R_{0}^{s}\left(\omega \right)-1]+\frac{M\eta N}{\mu }+\underset{\left(\alpha_{1},\alpha_{2}\right)\in R_{+}^{2}}{\sup}W\left(\alpha_{1},\alpha_{2}\right)+\frac{1}{2}\lambda_{1}\ln\varepsilon +\frac{1}{2}\lambda_{2}\ln\alpha_{2}\leq -1, \end{array}$$

(19) $$\begin{array}{cc} \; & -M\left(\beta +\mu \right)[R_{0}^{s}\left(\omega \right)-1]+\frac{M\eta N}{\mu }+\underset{\left(\alpha_{1},\alpha_{2}\right)\in R_{+}^{2}}{\sup}W\left(\alpha_{1},\alpha_{2}\right)+\frac{1}{2}\lambda_{1}\ln\alpha_{1}+\frac{1}{2}\lambda_{2}\ln\varepsilon \leq -1, \end{array}$$

(20) $$\begin{array}{cc} \; & -M\left(\beta +\mu \right)[R_{0}^{s}\left(\omega \right)-1]+\frac{M\eta N}{\mu }+\underset{\left(\alpha_{1},\alpha_{2}\right)\in R_{+}^{2}}{\sup}W\left(\alpha_{1},\alpha_{2}\right)+\frac{1}{2}\lambda_{1}\ln\alpha_{1}+\frac{1}{2}\lambda_{2}\ln\alpha_{2} \end{array}$$

$$\begin{array}{cc} \; & -\min\left\{\frac{\lambda_{1}\ln\frac{1}{\varepsilon }}{2\varepsilon },\frac{\lambda_{2}\ln\frac{1}{\varepsilon }}{2\varepsilon }\right\}\leq -1, \end{array}$$

(21) $$\begin{array}{cc} \; & -M\left(\beta +\mu \right)[R_{0}^{s}\left(\omega \right)-1]+M\eta \varepsilon +\underset{\left(\alpha_{1},\alpha_{2}\right)\in R_{+}^{2}}{\sup}W\left(\alpha_{1},\alpha_{2}\right)+\frac{1}{2}\lambda_{1}\ln\alpha_{1}+\frac{1}{2}\lambda_{2}\ln\alpha_{2}\leq -1, \end{array}$$

(22) $$\begin{array}{cc} \; & -M\left(\beta +\mu \right)[R_{0}^{s}\left(\omega \right)-1]+\frac{M\eta N}{\mu }+\underset{\left(\alpha_{1},\alpha_{2}\right)\in R_{+}^{2}}{\sup}W\left(\alpha_{1},\alpha_{2}\right)-\min\left\{\frac{N}{\varepsilon },\frac{\beta_{1}}{\varepsilon },\frac{\beta_{2}}{\varepsilon }\right\}\leq -1. \end{array}$$

Case1 If $\rho \in D_{\varepsilon }^{1}$, from (16) and (18), we have

$$\begin{array}{cc} G(\rho )\leq & -M\left(\beta +\mu \right)[R_{0}^{s}\left(\omega \right)-1]+\frac{M\eta N}{\mu }\\ \; & +\underset{\left(\alpha_{1},\alpha_{2}\right)\in R_{+}^{2}}{\sup}W\left(\alpha_{1},\alpha_{2}\right)+\frac{1}{2}\lambda_{1}\ln\alpha_{1}+\frac{1}{2}\lambda_{2}\ln\alpha_{2}\\ \; & \leq -M\left(\beta +\mu \right)[R_{0}^{s}\left(\omega \right)-1]+\frac{M\eta N}{\mu }+\\ \; & \underset{\left(\alpha_{1},\alpha_{2}\right)\in R_{+}^{2}}{\sup}W\left(\alpha_{1},\alpha_{2}\right)+\frac{1}{2}\lambda_{1}\ln\varepsilon +\frac{1}{2}\lambda_{2}\ln\alpha_{2}\leq -1. \end{array}$$

Case2 If $\rho \in D_{\varepsilon }^{2}$, from (16) and (19), we have

$$\begin{array}{cc} G(\rho )\leq & -M\left(\beta +\mu \right)[R_{0}^{s}\left(\omega \right)-1]+\frac{M\eta N}{\mu }\\ \; & +\underset{\left(\alpha_{1},\alpha_{2}\right)\in R_{+}^{2}}{\sup}W\left(\alpha_{1},\alpha_{2}\right)+\frac{1}{2}\lambda_{1}\ln\alpha_{1}+\frac{1}{2}\lambda_{2}\ln\alpha_{2}\\ \; & \leq -M\left(\beta +\mu \right)[R_{0}^{s}\left(\omega \right)-1]+\frac{M\eta N}{\mu }+\\ \; & \underset{\left(\alpha_{1},\alpha_{2}\right)\in R_{+}^{2}}{\sup}W\left(\alpha_{1},\alpha_{2}\right)+\frac{1}{2}\lambda_{1}\ln\alpha_{1}+\frac{1}{2}\lambda_{2}\ln\varepsilon \leq -1. \end{array}$$

Case3 If $\rho \in D_{\varepsilon }^{3}$, from (16) and (20), we have

$$\begin{array}{cc} G(\rho )\leq & -M\left(\beta +\mu \right)[R_{0}^{s}\left(\omega \right)-1]+\frac{M\eta N}{\mu }+\underset{\left(\alpha_{1},\alpha_{2}\right)\in R_{+}^{2}}{\sup}W\left(\alpha_{1},\alpha_{2}\right)\\ \; & +\frac{1}{2}\lambda_{1}\ln\alpha_{1}+\frac{1}{2}\lambda_{2}\ln\alpha_{2}-\frac{1}{2}\alpha_{1}\lambda_{1}\ln\alpha_{1}\\ \; & \leq -M\left(\beta +\mu \right)[R_{0}^{s}\left(\omega \right)-1]+\frac{M\eta N}{\mu }+\underset{\left(\alpha_{1},\alpha_{2}\right)\in R_{+}^{2}}{\sup}W\left(\alpha_{1},\alpha_{2}\right)\\ \; & +\frac{1}{2}\lambda_{1}\ln\alpha_{1}+\frac{1}{2}\lambda_{2}\ln\alpha_{2}-\frac{1}{2\varepsilon }\lambda_{1}ln\frac{1}{\varepsilon }\leq -1. \end{array}$$

Case4 If $\rho \in D_{\varepsilon }^{4}$, from (16) and (20), we have

$$\begin{array}{cc} G(\rho )\leq & -M\left(\beta +\mu \right)[R_{0}^{s}\left(\omega \right)-1]+\frac{M\eta N}{\mu }+\underset{\left(\alpha_{1},\alpha_{2}\right)\in R_{+}^{2}}{\sup}W\left(\alpha_{1},\alpha_{2}\right)\\ \; & +\frac{1}{2}\lambda_{1}\ln\alpha_{1}+\frac{1}{2}\lambda_{2}\ln\alpha_{2}-\frac{1}{2}\alpha_{2}\lambda_{2}\ln\alpha_{2}\\ \; & \leq -M\left(\beta +\mu \right)[R_{0}^{s}\left(\omega \right)-1]+\frac{M\eta N}{\mu }+\underset{\left(\alpha_{1},\alpha_{2}\right)\in R_{+}^{2}}{\sup}W\left(\alpha_{1},\alpha_{2}\right)\\ \; & +\frac{1}{2}\lambda_{1}\ln\alpha_{1}+\frac{1}{2}\lambda_{2}\ln\alpha_{2}-\frac{1}{2\varepsilon }\lambda_{2}ln\frac{1}{\varepsilon }\leq -1. \end{array}$$

Case5 If $\rho \in D_{\varepsilon }^{5}$, from (16) and (21), we have

$$\begin{array}{cc} G(\rho )\leq & -M\left(\beta +\mu \right)[R_{0}^{s}\left(\omega \right)-1]+\frac{M\eta N}{\mu }+\underset{\left(\alpha_{1},\alpha_{2}\right)\in R_{+}^{2}}{\sup}W\left(\alpha_{1},\alpha_{2}\right)\\ \; & +\frac{1}{2}\lambda_{1}\ln\alpha_{1}+\frac{1}{2}\lambda_{2}\ln\alpha_{2}\\ \; & \leq -M\left(\beta +\mu \right)[R_{0}^{s}\left(\omega \right)-1]+M\eta \varepsilon +\underset{\left(\alpha_{1},\alpha_{2}\right)\in R_{+}^{2}}{\sup}W\left(\alpha_{1},\alpha_{2}\right)\\ \; & +\frac{1}{2}\lambda_{1}\ln\alpha_{1}+\frac{1}{2}\lambda_{2}\ln\alpha_{2}\leq -1. \end{array}$$

Case6 If $\rho \in D_{\varepsilon }^{6}$, from (16) and (22), we have

$$\begin{array}{cc} G(\rho )\leq & -M\left(\beta +\mu \right)[R_{0}^{s}\left(\omega \right)-1]+\frac{M\eta N}{\mu }+\underset{\left(\alpha_{1},\alpha_{2}\right)\in R_{+}^{2}}{\sup}W\left(\alpha_{1},\alpha_{2}\right)\\ \; & +\frac{1}{2}\lambda_{1}\ln\alpha_{1}+\frac{1}{2}\lambda_{2}\ln\alpha_{2}-\frac{N}{I}\\ \; & \leq -M\left(\beta +\mu \right)[R_{0}^{s}\left(\omega \right)-1]+\frac{M\eta N}{\mu }+\underset{\left(\alpha_{1},\alpha_{2}\right)\in R_{+}^{2}}{\sup}W\left(\alpha_{1},\alpha_{2}\right)\\ \; & +\frac{1}{2}\lambda_{1}\ln\alpha_{1}+\frac{1}{2}\lambda_{2}\ln\alpha_{2}-\frac{N}{\varepsilon }\leq -1. \end{array}$$

Case7 If $\rho \in D_{\varepsilon }^{7}$, from (16) and (22), we have

$$\begin{array}{cc} G(\rho )\leq & -M\left(\beta +\mu \right)[R_{0}^{s}\left(\omega \right)-1]+\frac{M\eta N}{\mu }+\underset{\left(\alpha_{1},\alpha_{2}\right)\in R_{+}^{2}}{\sup}W\left(\alpha_{1},\alpha_{2}\right)\\ \; & +\frac{1}{2}\lambda_{1}\ln\alpha_{1}+\frac{1}{2}\lambda_{2}\ln\alpha_{2}-\frac{\beta_{2}E}{U}\\ \; & \leq -M\left(\beta +\mu \right)[R_{0}^{s}\left(\omega \right)-1]+\frac{M\eta N}{\mu }+\underset{\left(\alpha_{1},\alpha_{2}\right)\in R_{+}^{2}}{\sup}W\left(\alpha_{1},\alpha_{2}\right)\\ \; & +\frac{1}{2}\lambda_{1}\ln\alpha_{1}+\frac{1}{2}\lambda_{2}\ln\alpha_{2}-\frac{\beta_{2}}{\varepsilon }\leq -1. \end{array}$$

Case8 If $\rho \in D_{\varepsilon }^{8}$, from (16) and (22), we have

$$\begin{array}{cc} G(\rho )\leq & -M\left(\beta +\mu \right)[R_{0}^{s}\left(\omega \right)-1]+\frac{M\eta N}{\mu }+\underset{\left(\alpha_{1},\alpha_{2}\right)\in R_{+}^{2}}{\sup}W\left(\alpha_{1},\alpha_{2}\right)\\ \; & +\frac{1}{2}\lambda_{1}\ln\alpha_{1}+\frac{1}{2}\lambda_{2}\ln\alpha_{2}-\frac{\beta_{1}E}{S}\\ \; & \leq -M\left(\beta +\mu \right)[R_{0}^{s}\left(\omega \right)-1]+\frac{M\eta N}{\mu }+\underset{\left(\alpha_{1},\alpha_{2}\right)\in R_{+}^{2}}{\sup}W\left(\alpha_{1},\alpha_{2}\right)\\ \; & +\frac{1}{2}\lambda_{1}\ln\alpha_{1}+\frac{1}{2}\lambda_{2}\ln\alpha_{2}-\frac{\beta_{1}}{\varepsilon }\leq -1. \end{array}$$

From (17)–(22), we conclude that

$$G\left(\rho \right)\leq -1,\;\forall \rho \in \Omega \setminus D_{\varepsilon }.$$

There exists K>0 such that

(23) $$\begin{array}{c} G(\rho )\leq K<+\infty ,\;\forall \rho \in \Omega . \end{array}$$

For any initial value $(I\left(0\right),E\left(0\right),S\left(0\right),U\left(0\right),\alpha_{1}\left(0\right),\alpha_{2}\left(0\right))\in \Omega$ and t≥0, Taking the expectation of (17) after applying Itô’s integral yields:

(24) $$\begin{array}{c} \begin{array}{cc} 0 & \leq \frac{E[V\left(I\left(t\right),E\left(t\right),S\left(t\right),U\left(t\right),\alpha_{1}\left(t\right),\alpha_{2}\left(t\right)\right)]}{t}=\frac{E[V\left(I\left(0\right),E\left(0\right),S\left(0\right),U\left(0\right),\alpha_{1}\left(0\right),\alpha_{2}\left(0\right)\right)]}{t}\\ \; & \;+\frac{1}{t}\int_{0}^{t}E\left[LV\left(I\left(\varsigma \right),E\left(\varsigma \right),S\left(\varsigma \right),U\left(\varsigma \right),\alpha_{1}\left(\varsigma \right),\alpha_{2}\left(\varsigma \right)\right)\right]d\varsigma \\ \; & \leq \frac{E[V\left(I\left(0\right),E\left(0\right),S\left(0\right),U\left(0\right),\alpha_{1}\left(0\right),\alpha_{2}\left(0\right)\right)]}{t}\\ \; & \;+\frac{1}{t}\int_{0}^{t}E\left[G\left(I\left(\varsigma \right),E\left(\varsigma \right),S\left(\varsigma \right),U\left(\varsigma \right),\alpha_{1}\left(\varsigma \right),\alpha_{2}\left(\varsigma \right)\right)\right]d\varsigma +3M\sqrt[{3}]{c_{1}c_{3}\beta_{1}N}\\ \; & \;\frac{1}{t}\int_{0}^{t}E\left(\sqrt[{3}]{{\tilde{\alpha }}_{1}}-\sqrt[{3}]{\alpha_{1}\left(\varsigma \right)}\right)d\varsigma +3M\sqrt[{3}]{c_{2}c_{4}\beta_{2}N}\frac{1}{t}\int_{0}^{t}E\left(\sqrt[{3}]{\alpha_{2}}-\sqrt[{3}]{{\tilde{\alpha }}_{2}\left(\varsigma \right)}\right)d\varsigma \\ \; & \;+4M\sqrt[{4}]{c_{5}c_{6}c_{7}\theta \beta_{1}N}\frac{1}{t}\int_{0}^{t}E\left(\sqrt[{4}]{{\tilde{\alpha }}_{2}^{\prime }}-\sqrt[{4}]{\alpha_{2}\left(\varsigma \right)}\right)d\varsigma +\left(c_{1}+c_{2}+c_{7}\right)\frac{\omega N}{\mu }\\ \; & \;\frac{1}{t}\int_{0}^{t}E\left(\alpha_{1}^{2}\left(\varsigma \right)-\hat{\alpha_{1}}\right)d\varsigma +\left(c_{1}+c_{2}+c_{7}\right)\frac{\omega N}{\mu }\frac{1}{t}\int_{0}^{t}E\left(\alpha_{2}^{2}\left(\varsigma \right)-\hat{\alpha_{2}}\right)d\varsigma . \end{array} \end{array}$$

By Birkhoff’s ergodic theorem for the log-normal OU process, the time average converges almost surely to the stationary expectation. Since $\alpha_{i}\left(\infty \right)$ follows a log-normal distribution with $\ln\alpha_{i}\left(\infty \right)∼N\left(\ln{\bar{\alpha }}_{i},\sigma_{i}^{2}/\left(2\lambda_{i}\right)\right)$, we have for any k>0

$$E\left[\alpha_{i}^{k}\right]={\bar{\alpha }}_{i}^{k}e^{k^{2}\sigma_{i}^{2}/\left(4\lambda_{i}\right)}.$$

We obtain

$$\begin{array}{cc} \lim_{t\to \infty }\frac{1}{t}\int_{0}^{t}\alpha_{1}^{\frac{1}{3}}\left(\varsigma \right)\;d\varsigma \; & ={\bar{\alpha }}_{1}^{\frac{1}{3}}e^{\frac{\sigma_{1}^{2}}{36\lambda_{1}}}={\left(\hat{\alpha_{1}}\right)}^{\frac{1}{3}},\;\;\;\;\lim_{t\to \infty }\frac{1}{t}\int_{0}^{t}\alpha_{2}^{\frac{1}{3}}\left(\varsigma \right)\;d\varsigma ={\bar{\alpha }}_{2}^{\frac{1}{3}}e^{\frac{\sigma_{2}^{2}}{36\lambda_{2}}}={\left(\hat{\alpha_{2}}\right)}^{\frac{1}{3}}, \end{array}$$

(25) $$\begin{array}{cc} \lim_{t\to \infty }\frac{1}{t}\int_{0}^{t}\alpha_{2}^{\prime \frac{1}{4}}\left(\varsigma \right)\;d\varsigma \; & ={\bar{\alpha_{2}}}^{\prime \frac{1}{4}}e^{\frac{\sigma_{2}^{2}}{64\lambda_{2}}}={\left({\hat{\alpha_{2}}}^{\prime }\right)}^{\frac{1}{4}}. \end{array}$$

Taking the infimum on both sides of (24) and letting t→+∞, together with (25), we obtain

$$\begin{array}{cc} 0 & <\underset{t\to +\infty }{lim inf}\frac{1}{t}E\left[\int_{0}^{t}G\left(I\left(\varsigma \right),E\left(\varsigma \right),S\left(\varsigma \right),U\left(\varsigma \right),\alpha_{1}\left(\varsigma \right),\alpha_{2}\left(\varsigma \right)\right)\;d\varsigma \right]\\ \; & =\underset{t\to +\infty }{lim inf}\frac{1}{t}\int_{0}^{t}E\left[G\left(I\left(\varsigma \right),E\left(\varsigma \right),S\left(\varsigma \right),U\left(\varsigma \right),\alpha_{1}\left(\varsigma \right),\alpha_{2}\left(\varsigma \right)\right)\cdot 1_{\left\{\left(I\left(\varsigma \right),E\left(\varsigma \right),S\left(\varsigma \right),U\left(\varsigma \right),\alpha_{1}\left(\varsigma \right),\alpha_{2}\left(\varsigma \right)\right)\in D_{\varepsilon }\right\}}\right]d\varsigma \\ \; & \;+\underset{t\to +\infty }{lim inf}\frac{1}{t}\int_{0}^{t}E\left[G\left(I\left(\varsigma \right),E\left(\varsigma \right),S\left(\varsigma \right),U\left(\varsigma \right),\alpha_{1}\left(\varsigma \right),\alpha_{2}\left(\varsigma \right)\right)\cdot 1_{\left\{\left(I\left(\varsigma \right),E\left(\varsigma \right),S\left(\varsigma \right),U\left(\varsigma \right),\alpha_{1}\left(\varsigma \right),\alpha_{2}\left(\varsigma \right)\right)\in \Omega \setminus D_{\varepsilon }\right\}}\right]d\varsigma \\ \; & \leq K\underset{t\to +\infty }{lim inf}\frac{1}{t}\int_{0}^{t}P_{\left\{\left(I\left(\varsigma \right),E\left(\varsigma \right),S\left(\varsigma \right),U\left(\varsigma \right),\alpha_{1}\left(\varsigma \right),\alpha_{2}\left(\varsigma \right)\right)\in D_{\varepsilon }\right\}}d\varsigma \\ \; & -\underset{t\to +\infty }{lim inf}\frac{1}{t}\int_{0}^{t}P_{\left\{\left(I\left(\varsigma \right),E\left(\varsigma \right),S\left(\varsigma \right),U\left(\varsigma \right),\alpha_{1}\left(\varsigma \right),\alpha_{2}\left(\varsigma \right)\right)\in \Omega \setminus D_{\varepsilon }\right\}}d\varsigma \\ \; & \leq \left(K+1\right)\underset{t\to +\infty }{lim inf}\frac{1}{t}\int_{0}^{t}P_{\left\{\left(I\left(\varsigma \right),E\left(\varsigma \right),S\left(\varsigma \right),U\left(\varsigma \right),\alpha_{1}\left(\varsigma \right),\alpha_{2}\left(\varsigma \right)\right)\in D_{\varepsilon }\right\}}d\varsigma -1, \end{array}$$

which means

$$\underset{t\to +\infty }{lim inf}\frac{1}{t}\int_{0}^{t}P\left(\left(I\left(\varsigma \right),E\left(\varsigma \right),S\left(\varsigma \right),U\left(\varsigma \right),\alpha_{1}\left(\varsigma \right),\alpha_{2}\left(\varsigma \right)\right)\in D_{\varepsilon }\right)d\varsigma \geq \frac{1}{K+1}>0.\;a.s.$$

According to Lemma 2.1 [28], model (4) possesses at least one stationary probability distribution on Ω when $R_{0}^{s}>1$. The proof is completed.

□

**Remark** **2.** *According to Theorem 2, when $R_{0}^{s}>1$, the rumor will persist. The relationship between $R_{0}^{s}$ and $R_{0}$ satisfies*

- *$R_{0}^{s}\geq R_{0}$ for any $\sigma_{1},\sigma_{2}>0$, with equality if and only if $\sigma_{1}=\sigma_{2}=0$. Indeed, from ${\tilde{\alpha }}_{i}={\bar{\alpha }}_{i}e^{\sigma_{i}^{2}/\left(12\lambda_{i}\right)}\geq {\bar{\alpha }}_{i},$ with equality if and only if $\sigma_{i}=0$, and since $R_{0}$ is strictly increasing in $\alpha_{1},\alpha_{2}$ (all coefficients being positive), replacing ${\bar{\alpha }}_{i}$ by ${\tilde{\alpha }}_{i}$ in $R_{0}$ yields $R_{0}^{s}\geq R_{0}$.*
- *For specific reversion speeds $\lambda_{1},\lambda_{2}$ and volatility intensities $\sigma_{1},\sigma_{2}$, we may have $R_{0}<1<R_{0}^{s}$. This indicates that moderate noise can sustain rumor propagation even when the deterministic model predicts extinction.*

## 4. Extinction

Define the stochastic extinction threshold

$$\begin{array}{cc} R_{0}^{e}= & R_{0}+\frac{\left(\theta \beta_{2}+2\theta^{2}+\theta \mu \right)N\bar{\alpha_{2}}\left(1+\psi (\sigma_{2},\lambda_{2})\right)}{\mu \left(\theta +\mu \right)\min\left\{v_{1},v_{2},v_{3}\right\}\min\left\{\theta +\mu ,\frac{\mu (\theta +\mu )}{2\theta +\mu }\right\}}\\ \; & +\frac{R_{0}\left(\theta +\mu \right)\left[\bar{\alpha_{1}}\left(\psi \left(\sigma_{1},\lambda_{1}\right)+\mu \bar{\alpha_{2}}\psi \left(\sigma_{2},\lambda_{2}\right)\right)\right]}{\min\left\{\theta +\mu ,\frac{\mu (\theta +\mu )}{2\theta +\mu }\right\}}, \end{array}$$

where $\psi \left(\sigma_{i},\lambda_{i}\right)=\sqrt{1+e^{\frac{\sigma_{i}^{2}}{\lambda_{i}}}-2e^{\frac{\sigma_{i}^{2}}{4\lambda_{i}}}}$ characterizes noise intensity effects.

**Theorem** **3.** *If $R_{0}^{e}<1$, The rumor in model (4) will become extinct almost surely.*

**Proof.**  Define a $C^{2}$-Lyapunov function

$$P=v_{1}E+v_{2}S+v_{3}U\;\mathrm{with}\;v_{1}=R_{0},\;v_{2}=\frac{N}{\mu }\frac{{\bar{\alpha }}_{1}}{\theta +\mu },\;v_{3}=\frac{N}{\mu }\left[\frac{{\bar{\alpha }}_{2}}{\mu }+\frac{\theta {\bar{\alpha }}_{2}}{\mu (\theta +\mu )}\right].$$

When Itô’s formula is applied to lnP, one has

(26) $$\begin{array}{cc} L(\ln P) & =\frac{1}{P}[v_{1}\left(\alpha_{1}S+\alpha_{2}U\right)I-v_{1}\left(\beta +\mu \right)E+v_{2}\beta_{1}E-v_{2}\left(\theta +\mu \right)S\\ \; & \;+v_{3}\beta_{2}E+v_{3}\theta S-v_{3}\mu U]\\ \; & \leq \frac{1}{P}[R_{0}\left(\alpha_{1}S+\alpha_{2}U\right)I-R_{0}\left(\beta +\mu \right)E+\frac{N}{\mu }\frac{\alpha_{1}\beta_{1}}{\theta +\mu }E-\frac{N}{\mu }\alpha_{1}S\\ \; & \;+\frac{N}{\mu }\left(\frac{\alpha_{2}\beta_{2}}{\mu }+\frac{\theta +\alpha_{2}\beta_{2}}{\mu (\theta +\mu )}\right)E+\frac{N}{\mu }\left(\frac{\alpha_{2}\theta }{\mu }+\frac{\theta^{2}\alpha_{2}}{\mu (\theta +\mu )}\right)S-\frac{N}{\mu }\alpha_{2}U]\\ \; & \leq \frac{1}{v_{1}E+v_{2}S+v_{3}U}[\left({\bar{\alpha }}_{1}S\frac{N}{\mu }+{\bar{\alpha }}_{2}U\frac{N}{\mu }\right)\left(R_{0}-1\right)+\frac{N}{\mu }\frac{\theta \beta_{2}}{\mu (\theta +\mu )}{\bar{\alpha }}_{2}E\\ \; & \;+\frac{N}{\mu }\frac{2\theta^{2}+\theta \mu }{\mu (\theta +\mu )}{\bar{\alpha }}_{2}S]+\frac{N}{\mu }\frac{\theta \beta_{2}E+\left(2\theta^{2}+\theta \mu \right)S}{\mu \left(\theta +\mu \right)\min\left\{v_{1},v_{2},v_{3}\right\}\left(E+S+U\right)}\left|\alpha_{2}-{\bar{\alpha }}_{2}\right|\\ \; & \;+\frac{v_{1}}{v_{2}}\frac{N}{\mu }|\alpha_{1}-{\bar{\alpha }}_{1}|+\frac{v_{1}}{v_{3}}\frac{N}{\mu }\left|\alpha_{2}-{\bar{\alpha }}_{2}\right|\\ \; & \leq \min\left\{\frac{N{\bar{\alpha }}_{1}}{\mu v_{2}},\frac{N{\bar{\alpha }}_{2}}{\mu v_{3}}\right\}\left(R_{0}-1\right)+\frac{N}{\mu }\frac{\theta \beta_{2}+2\theta^{2}+\theta \mu }{\mu \left(\theta +\mu \right)\min\left\{v_{1},v_{2},v_{3}\right\}}{\bar{\alpha }}_{2}\\ \; & \;+\frac{N}{\mu }\frac{\theta \beta_{2}+2\theta^{2}+\theta \mu }{\mu \left(\theta +\mu \right)\min\left\{v_{1},v_{2},v_{3}\right\}}|\alpha_{2}-{\bar{\alpha }}_{2}|+\frac{v_{1}}{v_{2}}\frac{N}{\mu }|\alpha_{1}-{\bar{\alpha }}_{1}|+\frac{v_{1}}{v_{3}}\frac{N}{\mu }\left|\alpha_{2}-{\bar{\alpha }}_{2}\right|. \end{array}$$

When $R_{0}^{e}<1$, it follows that $R_{0}\leq R_{0}^{e}<1$. In this case, integrating (26) from 0 to *t* and dividing by *t* yields:

(27) $$\begin{array}{c} \begin{array}{cc} \frac{\ln P(t)-\ln P(0)}{t}\leq & \min\left\{\frac{N{\bar{\alpha }}_{1}}{\mu v_{2}},\frac{N{\bar{\alpha }}_{2}}{\mu v_{3}}\right\}\left(R_{0}-1\right)+\frac{N}{\mu }\frac{\theta \beta_{2}+2\theta^{2}+\theta \mu }{\mu \left(\theta +\mu \right)\min\left\{v_{1},v_{2},v_{3}\right\}}{\bar{\alpha }}_{2}\\ \; & +\frac{v_{1}}{v_{2}}\frac{N}{\mu }\left(\frac{1}{t}\int_{0}^{t}\left|\alpha_{1}\left(\tau \right)-{\bar{\alpha }}_{1}\right|d\tau \right)+\frac{v_{1}}{v_{3}}\frac{N}{\mu }\left(\frac{1}{t}\int_{0}^{t}\left|\alpha_{2}\left(\tau \right)-{\bar{\alpha }}_{2}\right|d\tau \right)\\ \; & +\frac{N}{\mu }\frac{\theta \beta_{2}+2\theta^{2}+\theta \mu }{\mu \left(\theta +\mu \right)\min\left\{v_{1},v_{2},v_{3}\right\}}\left(\frac{1}{t}\int_{0}^{t}\left|\alpha_{2}\left(\tau \right)-{\bar{\alpha }}_{2}\right|d\tau \right). \end{array} \end{array}$$

From [29], Cauchy–Schwarz and Birkhoff’s ergodic theorem for the log-normal OU process, we have

(28) $$\begin{array}{cc} \lim_{t\to \infty }\frac{1}{t}\int_{0}^{t}\left|\alpha_{i}\left(\tau \right)-{\bar{\alpha }}_{i}\right|\;d\tau & \leq \lim_{t\to \infty }{\left(\frac{1}{t}\int_{0}^{t}{\left(\alpha_{i}\left(\tau \right)-{\bar{\alpha }}_{i}\right)}^{2}\;d\tau \right)}^{1/2}\\ \; & ={\left(E\left[{\left(\alpha_{i}\left(\infty \right)-{\bar{\alpha }}_{i}\right)}^{2}\right]\right)}^{1/2}. \end{array}$$

Since $\ln\alpha_{i}\left(\infty \right)∼N\left(\ln{\bar{\alpha }}_{i},\sigma_{i}^{2}/\left(2\lambda_{i}\right)\right)$, the log-normal moments give

$$E\left[\alpha_{i}\left(\infty \right)\right]={\bar{\alpha }}_{i}e^{\sigma_{i}^{2}/\left(4\lambda_{i}\right)},\;E\left[\alpha_{i}^{2}\left(\infty \right)\right]={\bar{\alpha }}_{i}^{2}e^{\sigma_{i}^{2}/\lambda_{i}},$$

and hence

$$E\left[{\left(\alpha_{i}\left(\infty \right)-{\bar{\alpha }}_{i}\right)}^{2}\right]={\bar{\alpha }}_{i}^{2}\left(e^{\sigma_{i}^{2}/\lambda_{i}}-2e^{\sigma_{i}^{2}/\left(4\lambda_{i}\right)}+1\right).$$

Therefore

(29) $$\lim_{t\to \infty }\frac{1}{t}\int_{0}^{t}\left|\alpha_{i}\left(\tau \right)-{\bar{\alpha }}_{i}\right|\;d\tau \leq {\bar{\alpha }}_{i}\sqrt{e^{\sigma_{i}^{2}/\lambda_{i}}-2e^{\sigma_{i}^{2}/\left(4\lambda_{i}\right)}+1}\;\left(i=1,2\right),$$

Taking the superior limit in (27) and applying (28) gives

(30) $$\underset{t\to \infty }{lim sup}\frac{\ln P(t)}{t}\leq \min\left\{\theta +\mu ,\frac{\mu (\theta +\mu )}{2\theta +\mu }\right\}\left(R_{0}^{e}-1\right).$$

When $R_{0}^{e}<1$, the logarithmic decay implies

$$\lim_{t\to \infty }P\left(t\right)=0.\;$$

This completes the proof of rumor extinction. □

**Remark** **3.** *Theorems 2 and 3 reveal that stochastic disturbances have a dual effect on rumor spreading, characterized by two distinct thresholds: $R_{0}^{s}$ governs persistence, while $R_{0}^{e}$ governs extinction. Since $R_{0}^{s}\geq R_{0}$ (Remark 2) and $R_{0}^{e}\geq R_{0}$ (see the proof of Theorem 3), noise generally enhances persistence and hinders extinction. The dual effect is reflected in the fact that the noise intensities $\sigma_{1},\sigma_{2}$ and the mean-reversion speeds $\lambda_{1},\lambda_{2}$ act in opposite directions: increasing $\sigma_{1}$ or $\sigma_{2}$ raises both thresholds, whereas increasing $\lambda_{1}$ or $\lambda_{2}$ lowers them. Accordingly, reducing the noise intensities $\sigma_{1},\sigma_{2}$ or increasing the mean-reversion speeds $\lambda_{1},\lambda_{2}$ lowers both thresholds and thus facilitates rumor extinction. This suggests that stabilizing the information environment and expediting rebuttal efforts can effectively impede the further proliferation of rumors.*

## 5. Numerical Example

In this section, we will validate The primary theoretical findings of the proposed stochastic model through three numerical examples. All numerical simulations are performed using the Euler–Maruyama method for the stochastic system. The time step is fixed at h=0.01.

**Figure 1:**
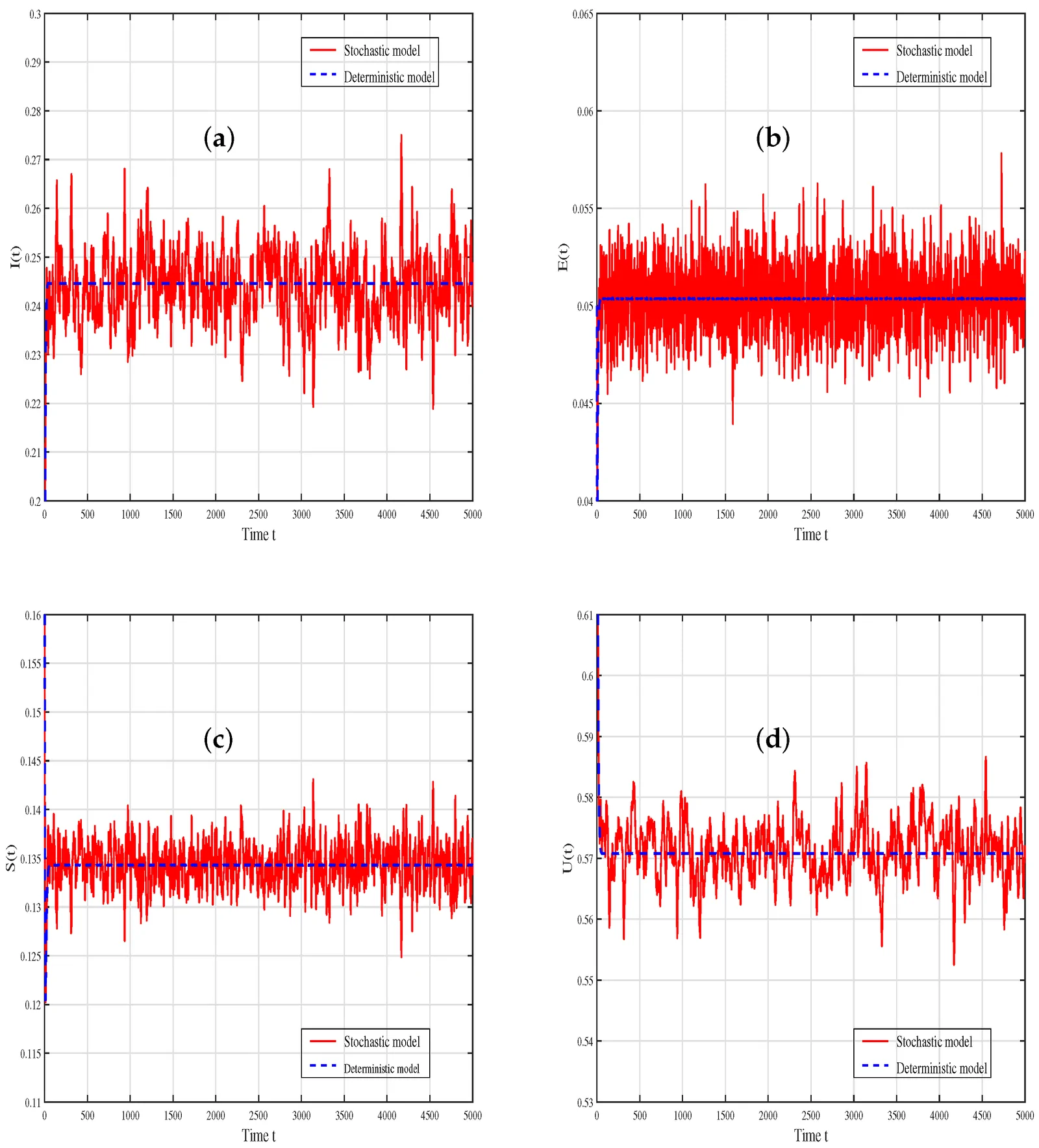
Movement tracks of (**a**) I(t), (**b**) E(t), (**c**) S(t), and (**d**) U(t) in the stochastic and deterministic models.

**Example** **1.** *The dynamical properties of the stochastic model (4) incorporating the bilinear incidence rate. Firstly, we fix the basic model parameters as follows: $N=0.05,\mu =0.05,\theta =0.1,\beta_{2}=0.3,$ $\beta_{1}=0.4,{\bar{\alpha }}_{1}=0.3,{\bar{\alpha }}_{2}=0.2,\sigma =0.05,\lambda_{i}=0.3\;\left(i=1,2\right)$. The basic reproduction number of model (4) can be calculated directly as $R_{0}^{s}\approx 4.0915>1$. The blue curves in Figure 1 demonstrate that the endemic equilibrium is asymptotically stable, meaning that the numbers of ignorant, hesitant, spreader, and opponent individuals converge to fixed values, leading to the persistence of the rumor.*

In contrast, the stochastic model (4) has threshold $R_{0}^{s}\approx 4.0915>1$. By Theorem 2, the system admits an ergodic stationary distribution, meaning that the rumor does not die out but instead fluctuates around a persistent level. This is illustrated by the red curves in Figure 1. Unlike the deterministic model, where the population sizes eventually settle at fixed values, the stochastic trajectories continue to exhibit random fluctuations over time. These fluctuations reflect the inherent uncertainty in real-world rumor transmission—such as variations in user activity, information overload, or external events—which cannot be captured by deterministic models.

**Figure 2:**
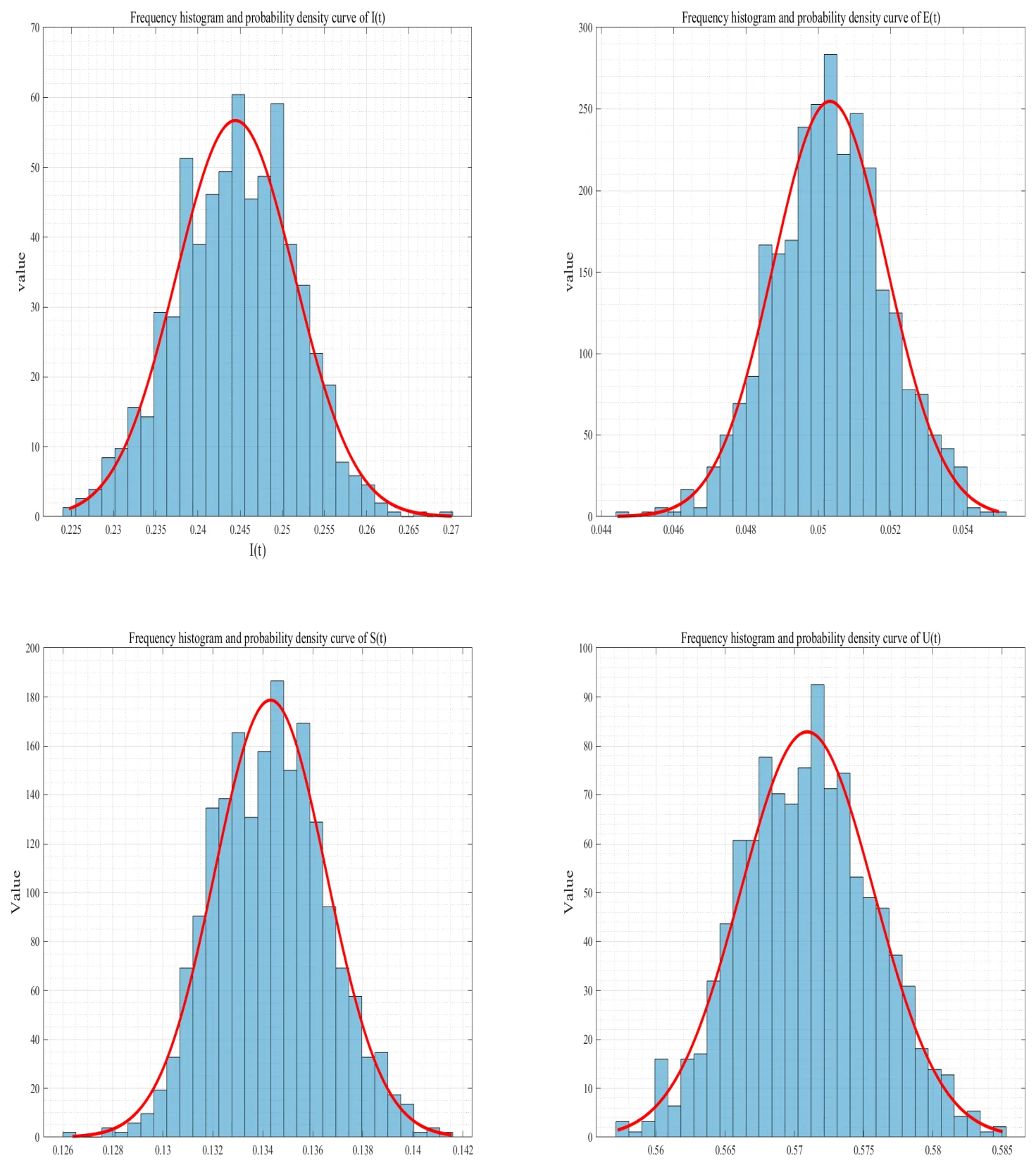
The marginal density function and frequency histogram for population.

To further quantify this variability, Figure 2 presents the marginal density functions of the stochastic model along with the corresponding frequency histograms obtained from simulations. The close agreement between the theoretical density and the simulated histogram validates our analytical results and provides a practical tool for predicting the likely range of rumor prevalence under different environmental noise levels. This information is valuable for policymakers: rather than relying on a single point estimate of rumor spread, they can assess the probability of extreme events and allocate resources accordingly.

**Figure 3:**
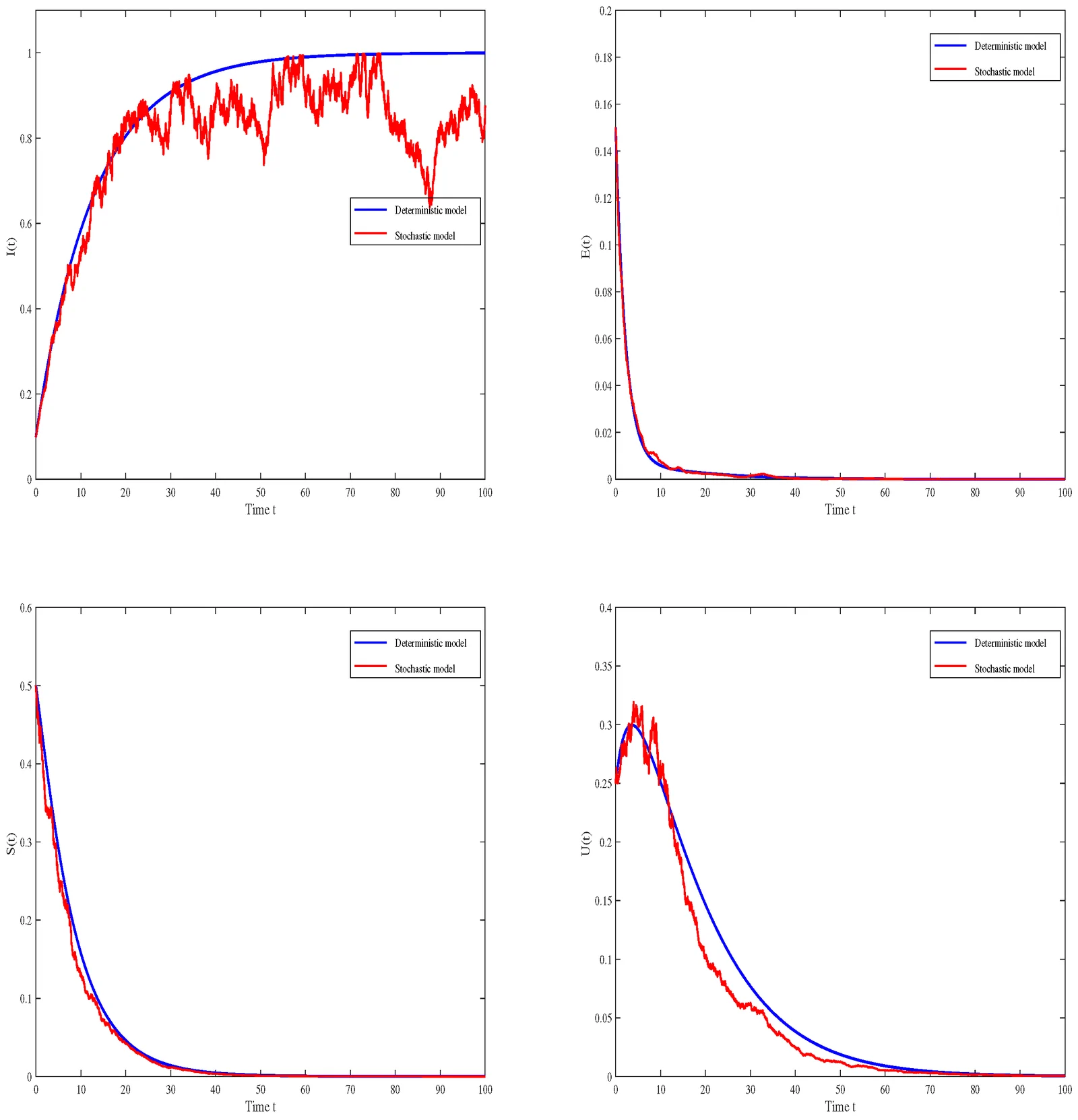
Variation curves of S(t),E(t),I(t),U(t) with the noise intensity σ=0.85,λ=0.5 which implies the extinction of the rumor in the long term.

**Figure 4:**
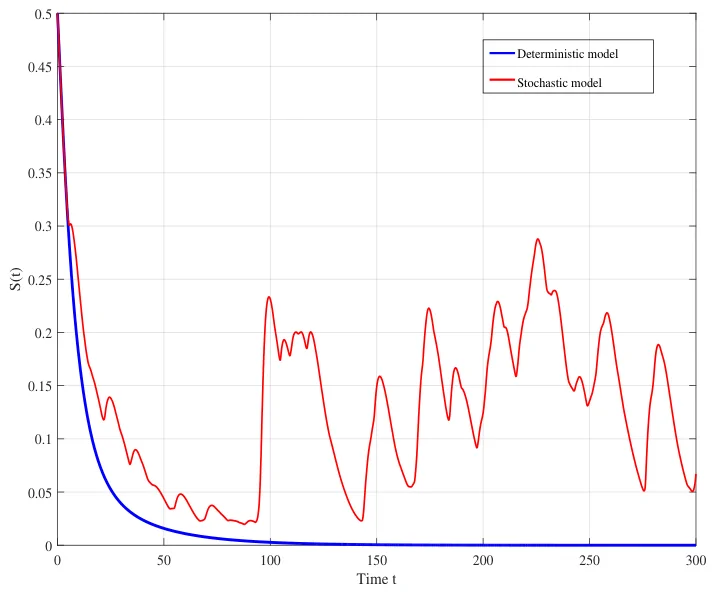
Noise-induced persistence. Time evolution of the spreader density S(t) for $R_{0}=0.5680<1$ and $R_{0}^{s}=2.8861>1$.

**Example** **2.** *We now investigate the extinction scenario by setting N=0.08,μ=0.08,θ=0.05, $\beta_{2}=0.2,\beta_{1}=0.15,\sigma_{i}=0.85,\lambda_{i}=0.5\;\left(i=1,2\right)$, we obtain $R_{0}^{e}=0.629301<1$. As shown in Figure 3, all population components E(t), S(t), U(t) decay to zero over time, confirming that the rumor eventually disappears almost surely. Compared with the deterministic model, the stochastic trajectory exhibits pronounced random oscillations and transient resurgences before decaying, rather than a monotone decline. This indicates that noise is not a mere perturbation superimposed on the deterministic solution, but can significantly alter the transient behavior of the rumor. This result is consistent with Theorem 3 and shows that when the stochastic threshold falls below unity, the combined effects of demographic factors and environmental fluctuations are sufficient to drive the rumor to extinction. More importantly, we further consider a regime where the two models predict different outcomes. Choosing parameters such that $R_{0}=0.5680<1<R_{0}^{s}=2.8861,$ the deterministic model predicts extinction, whereas the stochastic model admits a stationary distribution and the rumor persists. As shown in Figure 4, the deterministic trajectory decays to zero, while the stochastic trajectory remains bounded away from zero and fluctuates around a positive level. This confirms that moderate noise can sustain rumor propagation even when the deterministic threshold predicts extinction.*

**Example** **3.** *We analyze the normalized sensitivity indices of the extinction threshold $R_{0}^{e}$ with respect to each model parameter. The normalized sensitivity index of $R_{0}^{e}$ with respect to a parameter p is defined as*

$$S_{p}^{R_{0}^{e}}=\frac{\partial R_{0}^{e}}{\partial p}\cdot \frac{p}{R_{0}^{e}},$$

*which measures the relative change of $R_{0}^{e}$ induced by a small relative change of p. A positive value of $S_{p}^{R_{0}^{e}}$ indicates that increasing p promotes rumor persistence, while a negative value indicates that increasing p facilitates rumor extinction. Since the analytical expression of $R_{0}^{e}$ is highly nonlinear and difficult to differentiate explicitly, we conduct a numerical sensitivity analysis via the central difference method. All parameters are perturbed independently using the one-at-a-time (OAT) approach, i.e., when computing $S_{p}^{R_{0}^{e}}$, only p is varied while the remaining parameters are held at their baseline values. The baseline parameter values are the same as those in Example 2.*

**Figure 5:**
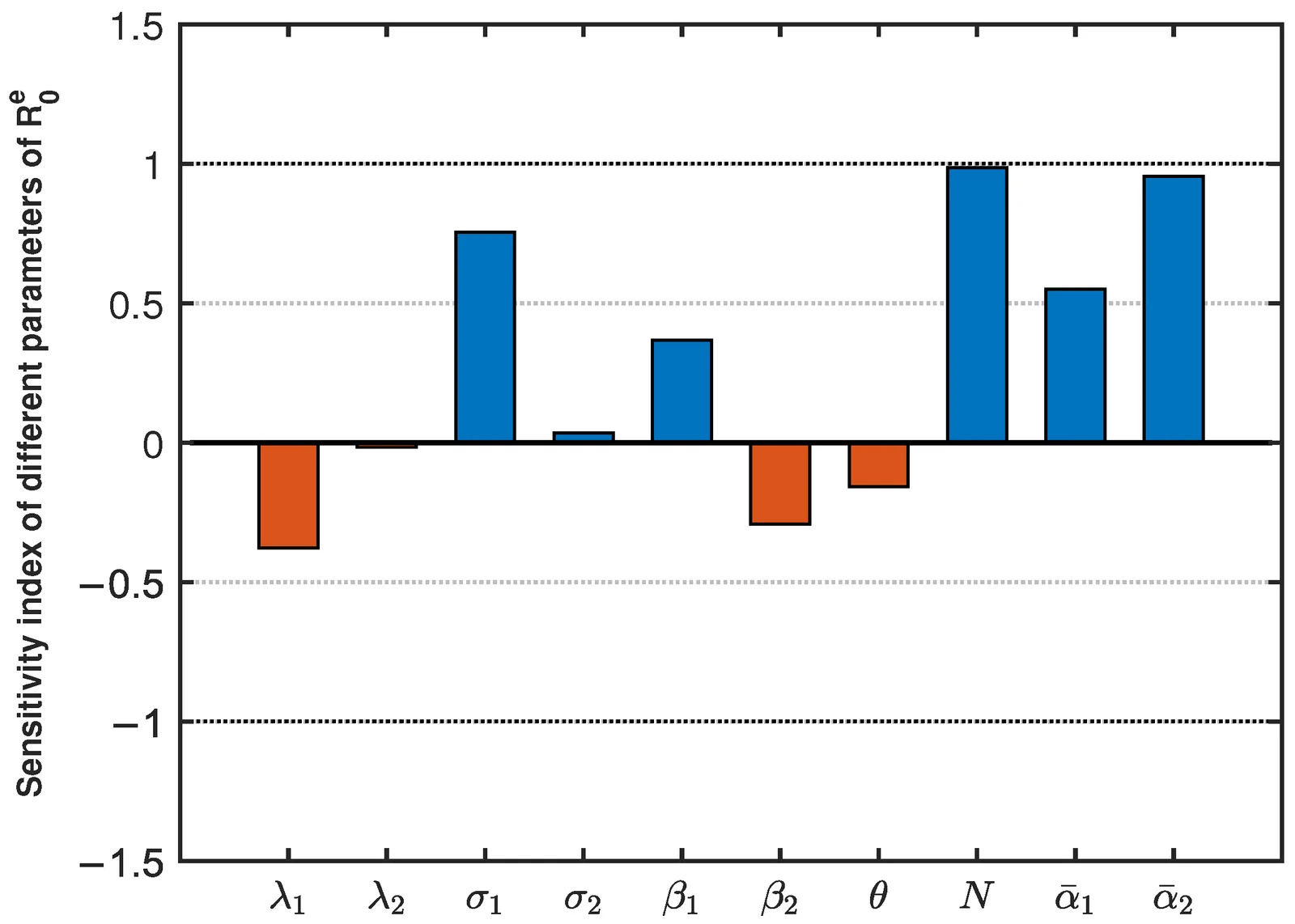
Sensitivity indices of $R_{0}^{e}$ against different parameters. Blue and orange bars represent positive and negative sensitivity indices, respectively.

The results are presented in Figure 5. Among all parameters, ${\bar{\alpha }}_{2}$ exhibits the strongest positive sensitivity, indicating that an increase in ${\bar{\alpha }}_{2}$ significantly elevates $R_{0}^{e}$, thereby making rumor extinction more difficult. The parameter *N* also shows a notable positive sensitivity, suggesting that a larger influx of susceptible individuals facilitates rumor persistence. In contrast, $\lambda_{1}$ and $\lambda_{2}$ (the mean-reversion speeds of the transmission rates) display negative sensitivities, implying that faster reversion to baseline levels suppresses rumor propagation and promotes extinction. The positive sensitivity of $\sigma_{1}$ indicates that increased volatility in the first transmission rate slightly enhances rumor persistence, while $\sigma_{2}$ shows a negligible positive effect.

From a practical perspective, these findings provide actionable insights for rumor control strategies. The positive correlation of *N* shows that public health authorities and social media platforms should prioritize interventions that limit the exposure of susceptible populations to unverified information. Moreover, the negative sensitivity of the mean-reversion rates $\lambda_{1}$ and $\lambda_{2}$ implies that accelerating the natural decline of transmission rates—for instance, through timely fact-checking, public education campaigns, or algorithmic content moderation—can effectively drive rumors toward extinction.

**Figure 6:**
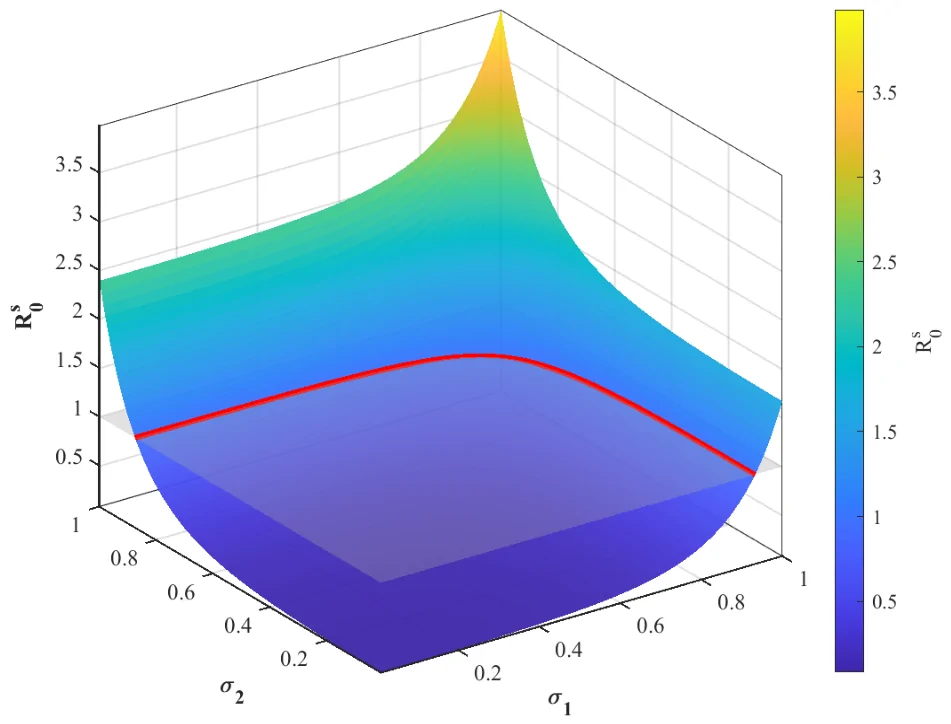
The threshold plane $R_{0}^{s}=1$ and the variation of $R_{0}^{s}$ with respect to the parameters $\sigma_{1}$ and $\sigma_{2}$. The red curve denotes the intersection $R_{0}^{s}=1$.

**Figure 7:**
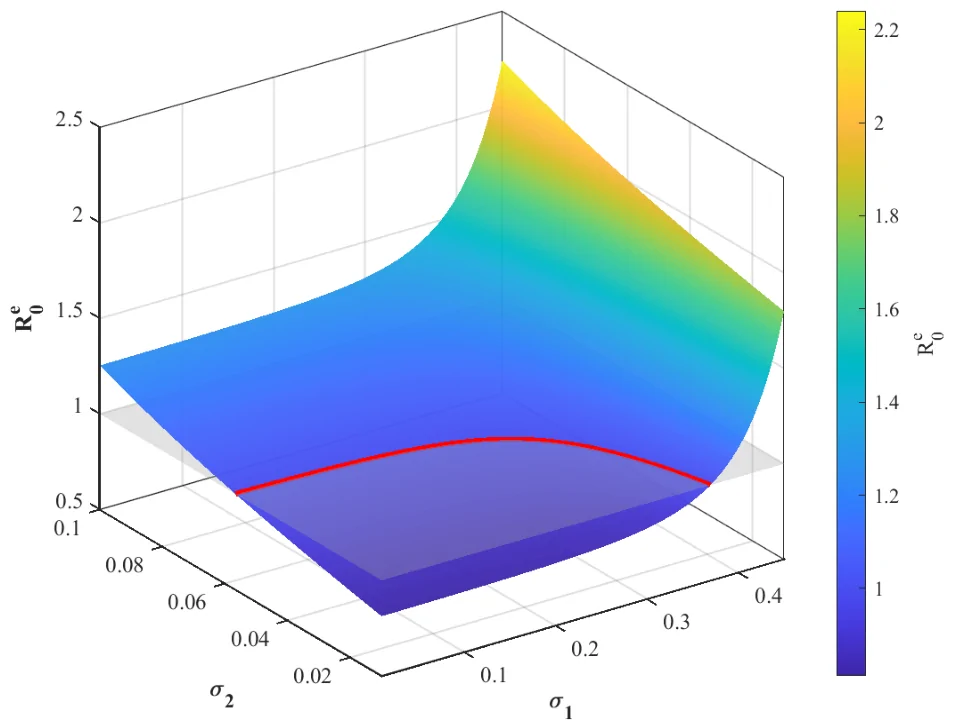
The threshold plane $R_{0}^{e}=1$ and the variation of $R_{0}^{e}$ with respect to the parameters $\sigma_{1}$ and $\sigma_{2}$. The red curve denotes the intersection $R_{0}^{e}=1$.

**Example** **4.** *To provide practical guidance for rumor control, we examine the dependence of the thresholds $R_{0}^{s}$ and $R_{0}^{e}$ on the noise intensities $\sigma_{1}$ and $\sigma_{2}$. We vary these parameters while fixing ${\bar{\alpha }}_{1}=0.015$, ${\bar{\alpha }}_{2}=0.01$, $\beta_{1}=0.02$, $\beta_{2}=0.01$, and keeping other biological parameters consistent with Example 1. Figure 6. visualizes the trend of $R_{0}^{s}$ as a function of $\left(\sigma_{1},\sigma_{2}\right)$. It is evident that $R_{0}^{s}$ increases with larger $\sigma_{1}$ and $\sigma_{2}$. The reference plane at $R_{0}^{s}=1$ clearly separates the persistence region $\left(R_{0}^{s}>1\right)$ from the extinction region $\left(R_{0}^{s}<1\right)$. This observation carries an important practical implication: higher noise intensity promotes rumor persistence. Intuitively, this means that in highly volatile information environments, rumors are more likely to persist. Figure 7. further illustrates the trend of $R_{0}^{e}$ with respect to $\left(\sigma_{1},\sigma_{2}\right)$ over a narrower parameter range. Notably, $R_{0}^{e}$ also increases as $\sigma_{1}$ and $\sigma_{2}$ increase. This suggests that reducing the fluctuation intensities can facilitate rumor extinction. These findings offer actionable strategies for rumor prevention and control: (i) stabilizing the information environment to reduce transmission-rate volatility, and (ii) implementing timely interventions, such as promptly releasing authoritative information to counteract rumors.*

## 6. Model Application

**Figure 8:**
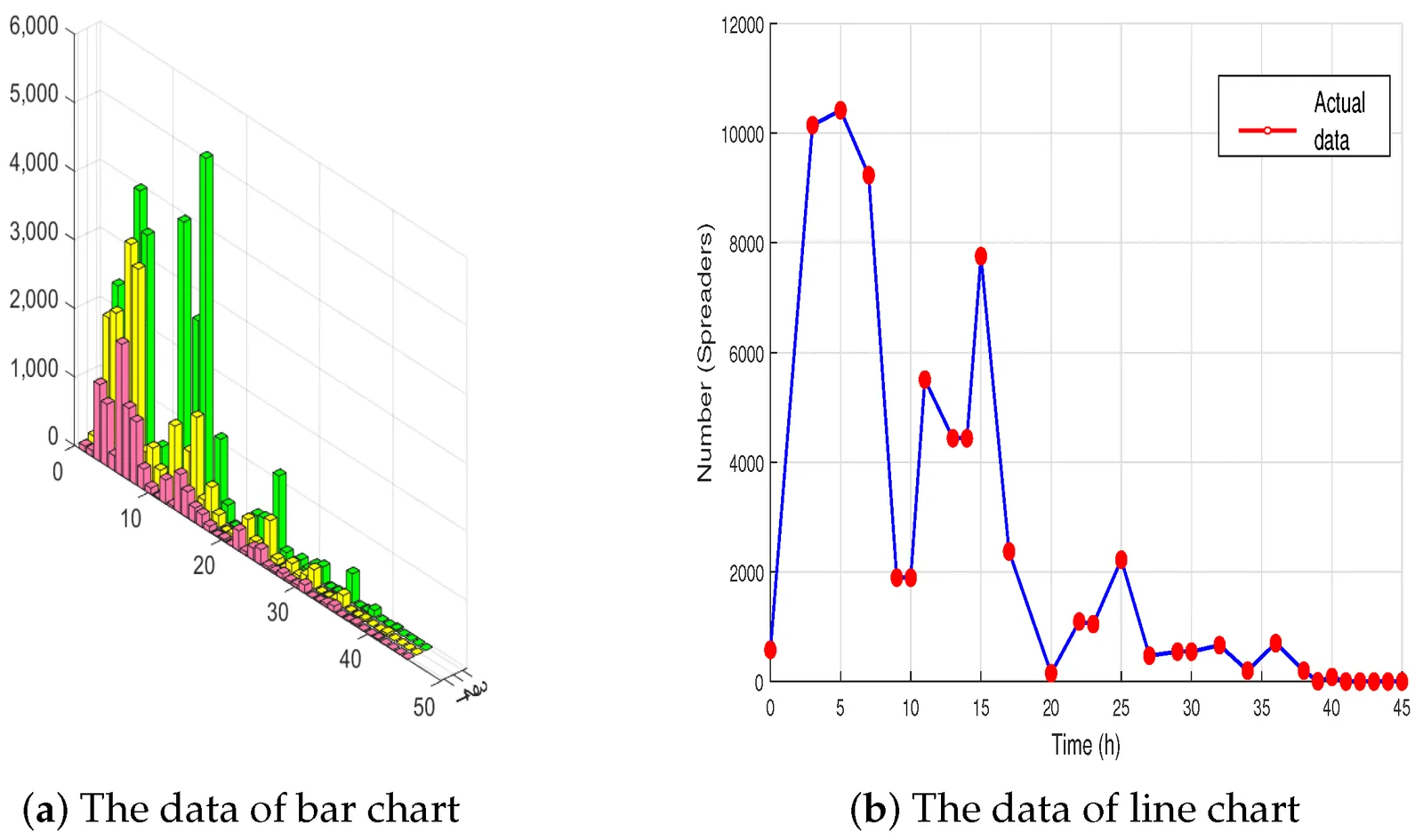
The number of reprints, comments and likes over time. Pink, yellow, and green bars represent $y_{1}$ (reprints), $z_{1}$ (comments), and $w_{1}$ (likes), respectively.

In this section, to further assess the practical relevance of our theoretical findings, we examine a real-world rumor event that occurred on the Chinese microblogging platform Weibo. On 22 December 2017, People’s Daily reported an incident titled “Chinese tourists in Thailand pull an elephant’s tail, enraging it; tour guide crushed to death while trying to save people.” This piece of news rapidly attracted public attention and triggered extensive online discussion. Leveraging the statistical data collected in [30], we plot in Figure 8a the histogram of the number of disseminators over time and in Figure 8b the temporal evolution of the number of comments and reposts at two-hour intervals within 42 h following the outbreak.

As clearly shown in Figure 8b, the number of disseminators exhibits a pronounced rise-and-fall pattern: it increases sharply in the early stage, reaches its peak around the 6th hour, and then gradually declines. This trend indicates that, in the absence of formal debunking, the rumor spread rapidly and maintained a high level of dissemination in the early stage. However, as the information aged and public interest waned, the propagation scale gradually diminished. Within our theoretical framework, this decay can be interpreted as a gradual decline in the effective transmission rate, which is jointly driven by a reduction in the noise intensity $\sigma_{i}$ and an increase in the mean-reversion speed $\lambda_{i}$ —reflecting the self-correcting nature of the information environment. Notably, it was not until the 42nd hour that the Thai police officially issued a debunking statement, which ultimately brought the rumor to a definitive end.

Based on the data collected in Table 1, the parameter values are set as follows: μ=0.01, β=0.5, $\beta_{1}=0.35$, $\beta_{2}=\beta -\beta_{1}=0.15$, θ=0.7, N=0.8. The fitting results are shown in Figure 8. In the figure, the spreader density curve derived from the Weibo platform data is represented by the red solid line, while the spreader density curve generated by our stochastic model (4) is represented by the blue solid line. We observe that the two curves exhibit qualitatively consistent trends: both rise rapidly in the early stage, then decline gradually, and eventually approach zero. This trend-level agreement suggests that the proposed stochastic model can reproduce the overall rise-and-fall pattern of a real rumor event, although the pointwise fit is limited by the strong hour-to-hour fluctuations of the observed data. A more complete validation would require additional data and a full likelihood-based estimation, which we leave for future work.

**Figure 9:**
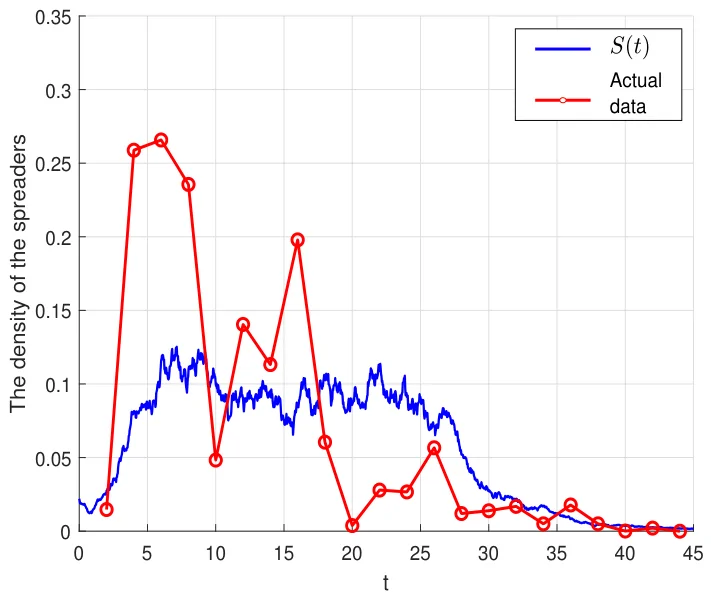
Comparison between the simulated spreader density S(t) (blue curve) and the normalized reprint counts from the Weibo data (red curve). The moderate pointwise $R^{2}$ reflects the strong hour-to-hour fluctuations of the observed series, while the model reproduces the overall rise-and-fall trend.

Since the available data provide only a single aggregate time series for the spreader compartment, a full likelihood-based estimation is not feasible. We therefore calibrate the effective transmission rate $\alpha_{\mathrm{avg}}=1.5$ by matching the simulated peak spreader density to the observed peak at t≈6 h, keeping all other parameters fixed at the values estimated from the Weibo data. The goodness of fit is quantified by $R_{\mathrm{smoothed}}^{2}=0.4106$ and RMSE=0.0563, computed against a 3-point moving-average smoothed version of the data. The moderate $R^{2}$ reflects the strong pointwise variability of the observed series (e.g., reprints jump from 89 at 2 h to 1117 at 3 h and then drop to 220 at 5 h), which a smooth four-compartment model cannot reproduce pointwise. The model nevertheless captures the overall rise-and-fall trend, as shown in Figure 9.

## 7. Conclusions

In this paper, we proposed a stochastic rumor propagation model that includes hesitant individuals, in which the propagation rate $\alpha_{i}$ follows the Ornstein–Uhlenbeck process. A major highlight of this study was that by introducing random perturbations, the inherent limitation of infinite variance caused by the time-varying parameter α in white noise perturbations was overcome. First, given any initial value, it is verified that the model always has a unique global positive solution. Second, the critical thresholds for rumor extinction and persistence were identified, respectively. Specifically, when $R_{0}^{e}\leq 1$, the rumor exhibited exponential extinction, whereas when $R_{0}^{s}\geq 1$, the model reached a steady-state distribution with persistent rumor presence. The derived threshold quantities $R_{0}^{s}$ and $R_{0}^{e}$ may be employed as quantitative metrics for rumor mitigation. As demonstrated by the sensitivity analyses in Figure 5 and Figure 6, decreasing the noise intensities $\sigma_{1}$ and $\sigma_{2}$ (while fixing $\lambda_{1},\lambda_{2}$) facilitates the reduction of $R_{0}^{s}$ to below one, thus promoting rumor extinction. Conversely, increasing the mean-reversion rates $\lambda_{1},\lambda_{2}$ yields the same extinction-facilitating effect. These results provide a mathematical rationale for social media operators to implement countermeasures, for instance, by moderating repost velocities and reinforcing content moderation protocols to reduce environmental noise. In future research, parameter estimation using real-world network data would be beneficial to assess the model’s predictive performance under practical conditions. Finally, numerical simulations validated the theoretical results. This study provided a robust theoretical foundation for rumor control.

The present model, as a first-step attempt, is developed under the assumption of a well-mixed population and does not incorporate spatial heterogeneity. We realize that this simplification may limit the applicability of our results to certain real-world scenarios, where geographical factors, network structures, or population mobility can significantly influence the spread of rumors. Therefore, we consider this a limitation of the current work. In our future research, we plan to extend the model by introducing spatial diffusion terms or adopting network-based frameworks to better capture the heterogeneous nature of information propagation. We hope that the findings of this study, despite this limitation, can serve as a useful foundation for further investigations that take spatial effects into account. 

## Figures and Tables

**Table 1:** Number of reprints, comments and likes.

Time (Hours) 1–11											
Time	1 h	2 h	3 h	4 h	5 h	6 h	7 h	8 h	9 h	10 h	11 h
Reprints	85	89	1117	902	220	1914	1050	919	299	92	20
Comments	163	69	2027	2165	839	3304	3014	418	539	285	24
Likes	111	60	2429	1506	719	4022	3444	390	490	189	71
**Time (Hours) 12–22**											
Time	12 h	13 h	14 h	15 h	16 h	17 h	18 h	19 h	20 h	21 h	22 h
Reprints	346	40	562	381	214	182	83	13	31	6	294
Comments	1067	126	831	1400	264	516	179	8	37	7	390
Likes	3978	206	2672	5114	385	1152	259	14	48	12	386
**Time (Hours) 23–33**											
Time	23 h	24 h	25 h	26 h	27 h	28 h	29 h	30 h	31 h	32 h	33 h
Reprints	57	176	226	44	34	79	39	50	119	4	20
Comments	135	108	580	83	19	161	52	124	280	2	17
Likes	412	158	1170	119	29	149	75	205	256	1	22
**Time (Hours) 34–44**											
Time	34 h	35 h	36 h	37 h	38 h	39 h	40 h	41 h	42 h	43 h	44 h
Reprints	48	86	3	13	30	2	2	22	0	0	0
Comments	43	180	1	8	27	0	0	36	0	0	0
Likes	47	428	1	22	97	0	0	24	0	0	0

## Data Availability

The authors confirm that the data supporting the findings of this study are available within the article.
